# Evidence for Electron Transfer between Graphene and Non‐Covalently Bound π‐Systems

**DOI:** 10.1002/chem.202000488

**Published:** 2020-04-17

**Authors:** Steffen M. Brülls, Valentina Cantatore, Zhenping Wang, Pui Lam Tam, Per Malmberg, Jessica Stubbe, Biprajit Sarkar, Itai Panas, Jerker Mårtensson, Siegfried Eigler

**Affiliations:** ^1^ Department of Chemistry and Chemical Engineering Chalmers University of Technology Kemivägen 10 41296 Gothenburg Sweden; ^2^ Institut für Chemie und Biochemie Freie Universität Berlin Takustraße 3 14195 Berlin Germany; ^3^ Department of Industrial and Materials Science Chalmers University of Technology Rännvägen 2A 41296 Gothenburg Sweden; ^4^ Institut für Chemie und Biochemie Freie Universität Berlin Fabeckstraße 34/36 14195 Berlin Germany; ^5^ Institut für Anorganische Chemie Universität Stuttgart Pfaffenwaldring 55 70569 Stuttgart Germany

**Keywords:** doping, functionalization, graphene, sensors, trication

## Abstract

Hybridizing graphene and molecules possess a high potential for developing materials for new applications. However, new methods to characterize such hybrids must be developed. Herein, the wet‐chemical non‐covalent functionalization of graphene with cationic π‐systems is presented and the interaction between graphene and the molecules is characterized in detail. A series of tricationic benzimidazolium salts with various steric demand and counterions was synthesized, characterized and used for the fabrication of graphene hybrids. Subsequently, the doping effects were studied. The molecules are adsorbed onto graphene and studied by Raman spectroscopy, XPS as well as ToF‐SIMS. The charged π‐systems show a p‐doping effect on the underlying graphene. Consequently, the tricationic molecules are reduced through a partial electron transfer process from graphene, a process which is accompanied by the loss of counterions. DFT calculations support this hypothesis and the strong p‐doping could be confirmed in fabricated monolayer graphene/hybrid FET devices. The results are the basis to develop sensor applications, which are based on analyte/molecule interactions and effects on doping.

## Graphical abstract

**Organic star‐shaped** tricationic molecules interact with graphene and a one‐electron transfer from graphene to the molecules leads to strong p‐doping.

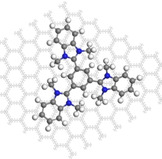

## Introduction

Graphene is part of growing interdisciplinary research, bridging natural sciences and material sciences.^1^ In a graphite crystal, several sheets of graphene are stacked on top of each other with an interlayer distance of 3.35 Å.^2^ Single graphene sheets, a two‐dimensional (2D) material, can be exfoliated from graphite. In analogy, other 2D materials can be produced by exfoliation. Up to now, graphene is the best studied 2D material.^3^ Graphene is characterized by its unique properties such as mechanical strength,^4^ electrical,^5^ optical and magnetic properties, which are linked to the symmetry of its crystal lattice. Its broad spectrum of properties allows graphene to be used in a series of applications, including amongst others high‐frequency transistors,^6^ high‐performance capacitors^,[7]^ transparent electrodes,^8^ the field of sensors^9^ and energy applications.^10^

Moreover, 2D materials can form van der Waals heterostructures, by stacking different 2D materials on top of each other by single layer flake transfer.^11^ These artificially assembled layered structures form systems analogous to pristine layered 2D materials.^12^ The resulting properties of stacks differ from those of the individual 2D materials. By using molecules, which are non‐covalently bound to 2D materials, the horizon of van der Waals heterostructures broadens,^13^ because the individual design of molecules leads to materials with desired electronic, optical and magnetic properties. The non‐covalent modification of graphene with aromatic molecules leading to either n‐ or p‐doping has been previously studied, however without focusing on the characterization of deposited molecules on the surface.^14^ Molecules affect the electronic properties of the 2D materials strongly,^15^ in contrast to adsorbed molecules on for example, graphite, where molecules change the interface properties, but not the bulk properties of graphite. The interface between the 2D materials and the molecules plays an important role in the performance of an electronic device.

**Figure 1:**
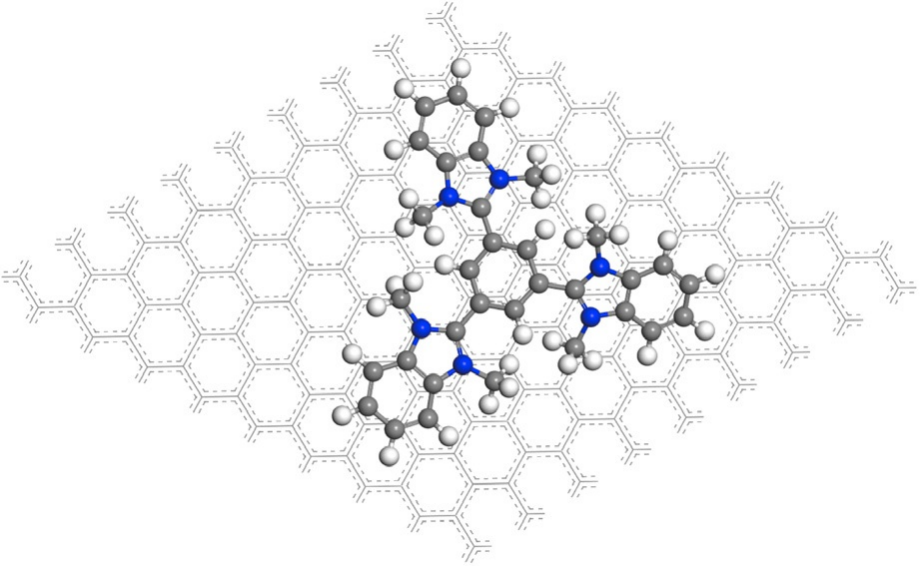
Optimized structure for tricationic molecule **6** (without counterions) non‐covalently bound to a 9×9 supercell of graphene in top‐view.

Although a plethora of 2D materials are currently in the focus of research and many more can be imagined to exist,^16^ the number of accessible molecules is orders of magnitude larger. The field of graphene organic hybrid electronics is growing,^17^ albeit not fully developed, and the materials produced are not extensively characterized. Here, we report the wet‐chemical non‐covalent functionalization of graphene using star‐shaped cationic N‐hetero‐π‐systems (Figure 1). These aromatic conjugated systems form a stable molecular layer on graphene. A partial electron transfer from graphene to the molecular layer results in p‐doping of the graphene. The results are confirmed by DFT calculations, AFM, XPS, ToF‐SIMS, cyclic voltammetry and electrical transport measurements.

## Results and Discussion

Star‐shaped cationic N‐hetero‐π‐systems were synthesized with different counterions, namely 1,3,5‐tris‐benzimidazolium benzene derivatives with I^−^, BF_4_^−^ and OTf^−^, respectively.

**Scheme 1:**
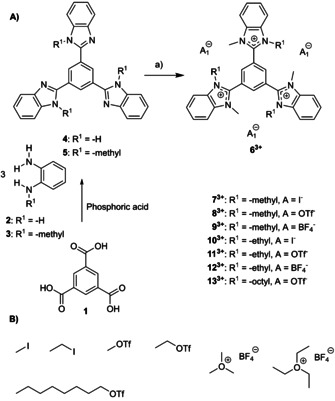
A) Synthesis of cationic molecules **6^3+^**–**13^3+^** from either 1,3,5‐tris (benzimidazolyl)benzene 4 or 1,3,5‐tris(N‐methyl benzimidazolyl)benzene 5 in the presence of various a) alkylation agents (3.3 equiv), acetonitrile, 24 h, room temperature. B) Alkylation agents to introduce methyl, ethyl, and octyl substituents with either iodide, tetrafluoroborate or triflate counterions.

As depicted in Scheme 1 A and described in the literature,^18^ trimesic acid **1** was condensed with either *o*‐phenyldiamine **2** or *N*‐methyl phenyldiamine **3** in phosphoric acid to yield 1,3,5‐tris(benzimidazolyl) benzene **4** or 1,3,5‐tris(*N*‐methyl‐benzimidazolyl) benzene **5**, respectively. Both compounds were converted into trications **6^3+^** through N‐quaternization using alkylation agents to introduce various substituents and counterions (Scheme 1 B).

The synthetic protocols and the characterization of all compounds shown in Scheme 1, and NMR and MS analyses are summarized in the Supporting Information (Figure S1–S23, Supporting Information). The obtained molecules (**7^3+^–13^3+^**, Scheme 1) were used to non‐covalently functionalize graphene. Both graphene prepared by CVD (chemical vapor deposition) method^19^ and graphene prepared by chemical reduction of oxo‐functionalized graphene (r‐oxo‐G) samples were used to enable reliable and complementary analyses. We introduced oxo‐G in recent years and the material differs from common graphene oxide by a more defined surface chemistry with oxo‐functional groups, such as epoxy‐, hydroxyl, carbonyl‐, carboxyl‐groups^20^ and by a controlled density of in‐plane lattice defects, by minimized over‐oxidation. The here used r‐oxo‐G is graphene with a density of lattice defects of about 0.5–0.8 %, according to statistical Raman spectroscopy.^21^ CVD graphene neither suffers from in‐plane vacancy defects nor oxo‐functional groups on the basal plane, although grain boundaries are present.^22^ Here we exploit the benefits of r‐oxo‐G and CVD graphene for analyses. Whereas oxo‐G is used for AFM, ToF‐SIMS (contrast by the shape of flakes) and the electrical transport measurements (ease of device fabrication), CVD graphene was used for Raman spectroscopy and XPS (complete coverage of the surface).

**Figure 2:**
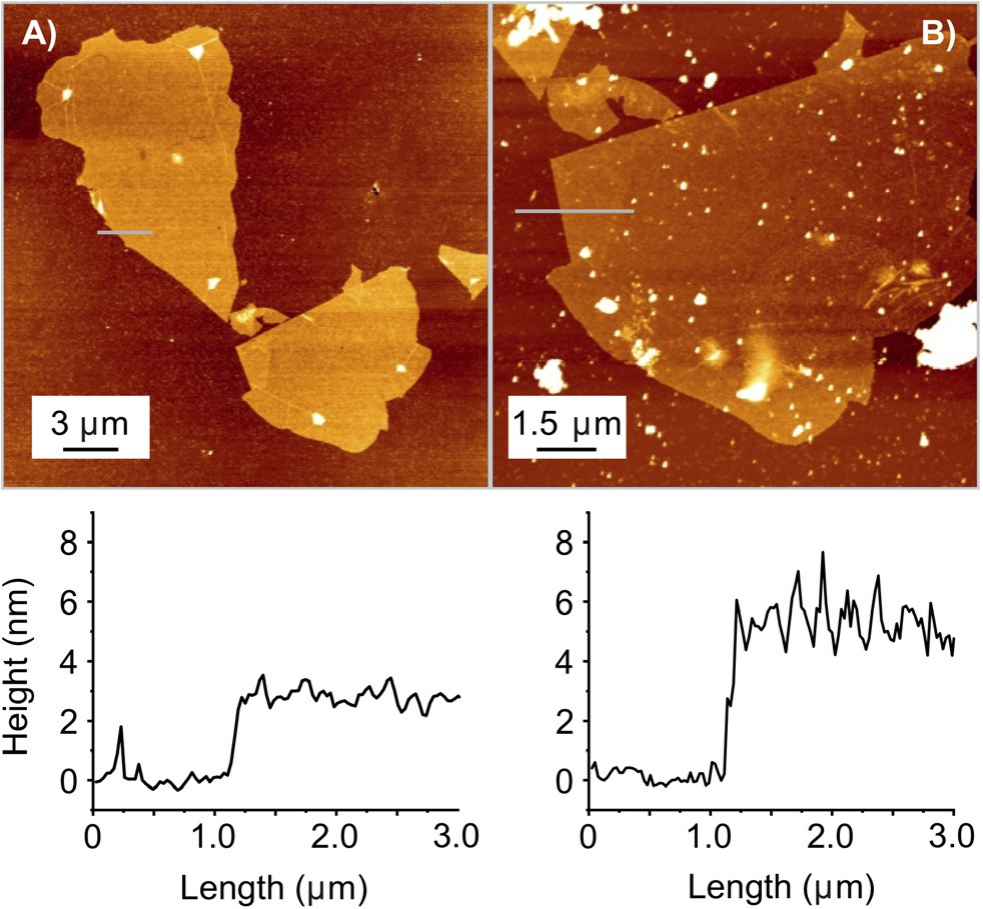
AFM image from r‐oxo‐G flake A) before and B) after functionalization with **8^3+^**. Height profiles along grey lines are shown below the AFM images.

The non‐covalent modification of graphene was accomplished by incubating a silicon wafer with deposited graphene, either CVD graphene or r‐oxo‐G, in a 12 mm methanol solution of the respective tricationic molecule for 2 hours at 4 °C. After incubation, the functionalized graphene wafer was removed from the incubation solution and rinsed with methanol to remove excess of the tricationic molecules. An overview of different sample preparations is summarized in the Supporting Information. To determine the topology of the functionalized graphene samples, AFM studies were performed (Figure 2). Before functionalization, the thickness of flakes of r‐oxo‐G is about 2.5 nm (Figure 2 A). After non‐covalent functionalization, the thickness of the r‐oxo‐G/hybrid increases to about 5 nm, as shown in the height‐profile of Figure 2 B. We note that the reference experiment using the uncharged compound **4** for non‐covalent functionalization of r‐oxo‐G also results in an increase of thickness from 2.5 nm to about 3.5 nm (Figure S41). Due to the polar nature of the benzimidazole moiety and hydrogen‐bond acceptor function, it seems likely that water or methanol molecules can be adsorbed under ambient conditions. Although some contaminants are visible on the surface of the flakes of the r‐oxo‐G/hybrid the roughness and measured height is overall homogeneous after functionalization. Thus, the AFM results indicate that molecules form indeed films and do not cluster to aggregates.

**Figure 3:**
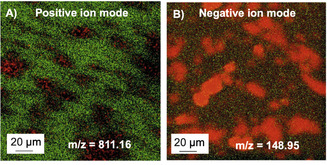
A) ToF‐SIMS image in positive ion mode showing the once reduced **8^3+^** on reduced oxo‐G on silicon substrate in a flake‐like pattern. Red: *m*/*z* 811.16, green: Si+. B) ToF‐SIMS image in negative mode showing the triflate anion from **8^3+^** on reduced oxo‐G on silicon substrate in a flake‐like pattern. Red: *m*/*z* 148.95, green: Si−.

The existence of the tricationic molecules on the surface of r‐oxo‐G/hybrid was further studied by time‐of‐flight secondary ion mass spectrometry (ToF‐SIMS) analysis. The adsorbed tricationic molecules can be identified by ToF‐SIMS analysis. An area of 150 μm *x* 150 μm of a film of flakes of r‐oxo‐G/**8^3+^** (notation: material on bottom and molecules on top) was scanned in positive mode. The most prominent signal observed was *m/z* 811.16, which can be related to **8^3+^** through the loss of one of its counterions. As shown in Figure 3 A (red color), the flake‐like pattern is revealed. The appearance of the *m*/*z* signal all over the flake with almost constant intensity confirms the homogeneous non‐covalent functionalization of the r‐oxo‐G. In addition, no signal is found on the bare SiO_2_ surface, indicating that molecules are only adsorbed on the r‐oxo‐G flake.

Thus, these observations corroborate the height profiles obtained by AFM measurements. Also, although with lower signal intensity, signals were observed at *m/z* 662.73 and *m/z* 513.28 corresponding to the mass of the trication without 2 and 3 counterions, respectively, (Figure S32–S36). In addition, ToF‐SIMS was conducted in negative ion mode to detect the triflate counterion with *m/z* 148.95. The map of Figure 3 B depicts also that triflate ions are located on the r‐oxo‐G flakes (red color). Reference experiments with CVD graphene as substrate show the non‐covalent functionalization of the complete sample area (Figure S37**)**. However, experiments with a blank Si/SiO_2_ wafer as substrate reveals that the tricationic molecules are not adhering to the SiO_2_ surface (Figure S38). A Si/SiO_2_ wafer incubated in methanol also did not show signals from the trication molecules **(**Figure S39). An optical image of a functionalized r‐oxo‐G sample is included in the Supporting Information (Figure S40).

**Figure 4:**
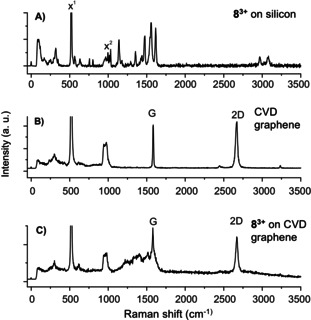
Raman spectra of A) tricationic molecule **8^3+^**, B) CVD graphene and C) trication molecule **8^3+^** on CVD graphene. As substrate for all samples silicon wafer (300 nm of SiO_2_ on surface) were used showing at x^1^=520 cm^−1^ the signal for crystalline silicon and at x^2^=900–1000 cm^−1^ the signal for amorphous silicon dioxide on the silicon surface.^26^ The signal intensities from x^1^ are not fully shown.

Raman spectra were recorded to monitor signatures of the molecules on SiO_2_ surface and on graphene surface.^23^ The Raman spectra of 1,3,5‐tris(*N*‐methylbenzimidazolyl) benzene **8^3+^** on SiO_2_ substrate (Figure 4 A) show aromatic C−H bond vibrations in the region between 2980–3020 cm−1, C=N bond vibrations between 1610–1680 cm^−1^ and aromatic ring vibrations between 1450–1610 cm^−1^. The bond vibrational signals at 1170–1410 cm^−1^ and between 670–780 cm^−1^ stem from the C−F and C−S bonds, respectively, in the triflate counterions.^24^ Neat graphene shows two distinct Raman peaks, the G peak at about 1584 cm^−1^ and the 2D peak at around 2700 cm^−1^ (Figure 4 B, Figure S24).^25^

For this study, CVD graphene was used because its Raman peaks are sharper compared to the peaks for r‐oxo‐G. Thus, the choice of CVD graphene reduced the interfering overlap with other peaks in the subsequent analysis. Raman spectra from non‐covalently functionalized graphene show the presence of broad and overlapping signals between 1200–1600 cm^−1^_,_ which stem from molecule **8^3+^** (Figure 4 C). We propose that the broadening of the molecular peaks is a consequence of the interaction with graphene. Raman spectra for the systems composed of trication **8^3+^** (without counterions) or the reduced form **8^2+^**, (dicationic species, without counter ions) on graphene computed on the density functional theory (DFT) level of theory support this interpretation (Figure S44). Reference Raman measurements on r‐oxo‐G/**8^3+^** indicate, in agreement with the ToF‐SIMS results, that molecules are only adsorbed on the graphene flakes and not on the bare SiO_2_. Raman spectra recorded on CVD graphene and CVD graphene/**8^3+^** reveals that **8^3+^** is adsorbed on graphene. Analysis of the shift of the position of the G peak, which is sensitive to doping,^27^ is not reliable due to overlapping signals. Instead, the 2D peak is analyzed statistically for CVD graphene and CVD graphene/**8^3+^**, respectively (Figure S26). The influence of the solvent methanol on CVD graphene was investigated and did not show any significant influence on the G and 2D band (Figure S27). XPS was used to analyze the composition of the surface of r‐oxo‐G/hybrid materials.^28^ In accordance with the fact that the graphene lattice contains only chemically equivalent carbon (C), the XPS spectrum of pristine graphene on silicon substrate shows a C 1s peak centered at 284.6 eV (Figure S28).^29^ The observed binding energy is consistent with the sp^2^‐hybridized character of the carbon present in graphene, together with minor contribution from certain oxidized states that cannot be completely avoided during the preparation_._ 29, 30 Other observed signals correspond to silicon (Si) and oxygen (O), at 104 eV and 533 eV, respectively,^31^ originating from the SiO_2_‐intersurface in between the graphene layer and silicon substrate. The nitrogen (N) signal observed at 400 eV,^31^ likely corresponding to adsorbed molecular nitrogen, is very weak in the case of silicon substrate and is expected to give negligible effects on the latter molecular analysis (Figure S29). Still, this element is of interest for the analysis of those graphene hybrids (i.e., the molecular adsorbates) that contain nitrogen. Depending on the kind of counterion, the adsorbed materials also contain either iodine (I), (i.e., **7^3+^**), oxygen (O) and sulfur (S), (i.e., **8^3+^**, **11^3+^**) or boron (B) and fluorine (F) (i.e., **9^3+^**, **12^3+^**). Comparison among the XPS spectra of pristine graphene, functionalized graphene and the neat molecules on silicon confirms the non‐covalent adsorption of the molecular systems on graphene. XPS samples with the neat molecules on substrates were prepared by drop casting a methanol solution of the respective tricationic molecule on a silicon wafer. The preparation of non‐covalently modified graphene was described previously.

**Figure 5:**
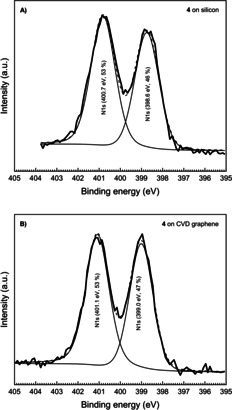
High resolution scan at the N 1s region for A) neutral **4** on CVD graphene substrate and B) **4** on silicon substrate.

**Figure 6:**
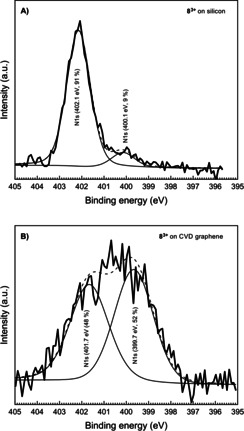
High resolution scan at the N 1s region for A) **8^3+^** on silicon substrate and B) **8^3+^** on CVD graphene substrate.

The XPS spectrum of the neat neutral molecule **4** on silicon shows two binding energies for nitrogen at 398.6 eV and 400.5 eV (Figure 5 A), respectively. This corresponds to the two chemically different nitrogen atoms in this molecule, that is, an imine type of nitrogen (R_2_C=NR) and a secondary amine type of nitrogen atom (R_2_‐NH). The binding energies for these two nitrogen atoms increase slightly to 399.0 eV and 401.0 eV, respectively, when the molecule **4** is adsorbed on graphene (Figure 5 B). This implies that the electron density on the neutral molecule is less when adsorbed on CVD graphene as compared to when it is adsorbed on the silicon wafer. This leads to a stronger electron attraction within the molecule chemisorbed on CVD graphene. The corresponding carbon peak position has also increased from 284.7 eV to 285.1 eV when the substrate is CVD graphene (Figure S29). The tricationic molecule **8^3+^** however shows a different behavior when non‐covalently bound to graphene. Due to quaternization, only one N 1s signal is expected, that is, all six nitrogen atoms are chemically equivalent. The XPS data recorded for the tricationic molecule **8^3+^** on silicon agrees with this picture (Figure 6 A**)**. It shows one major peak at the binding energy 402.1 eV, consistent with imidazolium nitrogen atoms.^32^ A second peak of much lower intensity at 400.1 eV is also observed but so far unaccounted for.

As this minor peak is also present in the XPS spectra of tricationic molecules **7^3+^** and **11^3+^** on silicon, it could possibly correspond to an ensemble of tricationic molecules binding to the silicon surface in a distinctly different mode compared to the vast majority. On the other hand, the XPS spectrum obtained for the sample with the tricationic molecule **8^3+^** on CVD graphene shows one very broad signal that can be fitted by two components of equal intensity (Figure 6 B**)** what is consistent with the presence of two sets of nitrogen atoms. XPS analysis of the N 1s components reveals two species. Accordingly, the signal at around 399.7 eV corresponds to an iminium type state and the other component located around 401.7 eV agrees with the expected value for an imidazolium nitrogen state.

As will be discussed later, the two components may correspond to two various nitrogen atoms either facing or pointing away from the graphene. The change in the ratio of the N 1s signals also gives information about the doping of graphene by non‐covalently bound molecules. Both p‐33 and n‐doping^34^ of non‐covalently bound molecules on graphene has been shown previously with XPS studies. Doping or charge transfer from graphene to the molecule results in an expanded delocalization of the positive charge into the valence band of graphene and reduced charge density on the molecule. This decreases the coulomb attraction between the anionic counterions and the cationic molecules, which potentially could be observed as an increase in trication to counterion ratio. Thus, we determined the ratios between the trications (i.e., N) and counterions (i.e., I, O, S, B and F) in the samples. The XPS spectrum recorded for trication **8^3+^** on silicon gives a ratio among fluorine, nitrogen and sulfur (F/N/S) of 3:2:1. This is in agreement with an assembly of one trication and three counterions, which corresponds to the molecular formula C_36_H_33_F_9_N_6_O_9_S_3_. After non‐covalent binding of **8^3+^** to graphene, this ratio changes to 1:3:0. The signal for the sulfur atoms is below the detection limit. The decrease of fluorine and sulfur, which originate from the triflate counterion, indicates that the triflate counterions become more exchangeable and possibly even superfluous.

**Figure 7:**
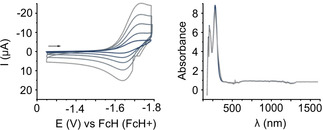
A) Cyclic voltammogram of first reduction peak from **12^3+^** at *E*_red,1_=−1.65 V) (different scan rates 25–1000 mVs^−1^) in ACN with 0.1 m (Bu_4_NPF_6_) vs. FcH/FcH^+^. B) UV/Vis‐NIR spectroelectrochemistry of **12^3+^** in ACN showing both, the absorption spectrum of the native species of **12^3+^** (blue) and of the reoxidized species of **12^+3^** (grey).

The XPS data recorded for **7^3+^** and **11^3+^** on CVD graphene show similar trends (Figures S30 and S31). Charge transfer from graphene to the non‐covalently bound molecules could explain this observation. Due to the weaker Coulomb attraction, some of the counter ions could potentially be removed by rinsing the sample with methanol during the sample preparation, or exchanged by some negatively charged species in the methanol solvent, for example, methoxide ions. A summary of all tested molecules is depicted in Scheme 1 (on silicon and on graphene) with their respective binding energies and the elemental content of certain elements is shown in Table 1. For all tested tricationic molecules, the elements which are present in the counterions are decreased when adsorbed on CVD graphene. The redox properties of **9^3+^** and **12^3+^** were investigated by cyclic voltammetry in acetonitrile (Figure S45, S46). Both compounds show several reduction events. The first one‐electron reduction of **12^3+^** corresponds to a reversible one‐electron transfer at *E*_red,1_=−1.65 V (Figure 7 A). These results show that the tricationic molecules are stable after receiving one electron. To support these observations, in situ UV/Vis‐NIR spectroelectrochemistry with **12^3+^** in acetonitrile was performed. The native form of **12^3+^** displays its main absorption bands in the UV region. On one‐electron reduction several long wavelength bands in the visible and the NIR region appear (Figure S47). Such long wavelength bands are usually a sign for the generation of radical species. Figure 7 B shows both, the absorption spectrum for the native **12^3+^** and for the reoxidized species of **12^3+^**. The uniform overlap of both spectra indicates that the same species was analyzed and that the first reduction event is reversible.

**Table 1:** Overview of binding energies of the N 1s peaks from all with XPS studied neutral and tricationic molecules and their graphene/hybrids.

Sample on substrate	Nitrogen content [at. %]	Relative intensity [%]				Atomic ratio
		Binding energy [eV]				
		398.5–399.0	399.5–400.0	400.5–401.0	401.5–402.0	
		Imine^[a]^	Iminium	Secondary amine^[a]^	Imidazolium^+[a]^	
**4**						
Si	13.5	45	–	55	–	(n.a.)
CVD	10.5	47	–	53	–	(n.a)
						
**7^3+^**						N/I
Si	5.2	–	12	–	88	5:4
CVD	2.2	–	41	–	59	7:1
						
**8^3+^**						F/N/S
Si	6.5	–	9	–	91	3:2:1
CVD	3.0	–	52	–	48	1:3:0
						
**11^3+^**						F/N/S
Si	8.2	–	4	–	96	3:2:1
CVD	4.2	–	63	–	37	1:3:0

[a] Ref. 32.

**Figure 8:**
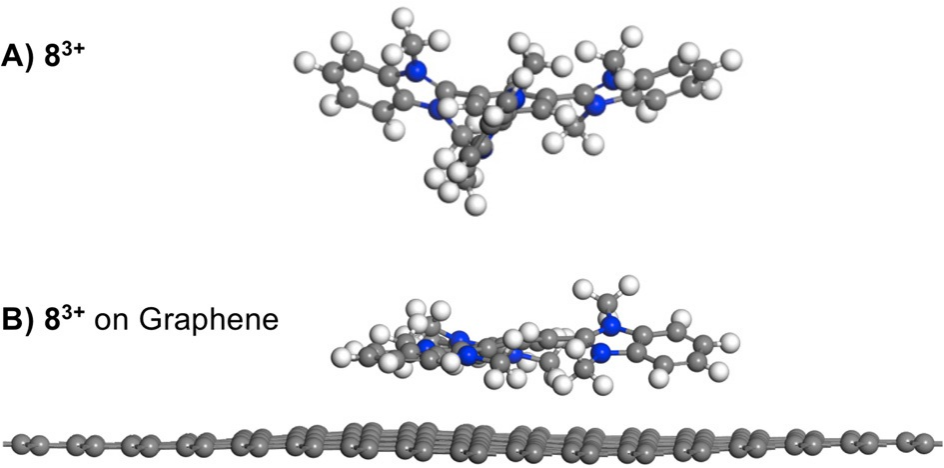
Optimized geometry of A) tricationic **8^3+^** (without counterions) and B) **8^3+^** non‐covalent bound to graphene.

For the purpose of rationalizing the experimental observations, computational quantum mechanical modelling was performed to gain insight into the interaction between tricationic molecules and graphene. A single molecule of **8^3+^** (without counterions) on top of a 9×9 supercell of graphene served as the unit cell in our model system. Density functional theory with periodic boundary calculations was used to study the overall electron distribution in the system. The optimized geometry of **8^3+^** alone at the same level of sophistication is propeller shaped, characterized by an average dihedral angle of 53.2° (44.7°, 55.9°, 59.02°) between the central benzene ring and the appended benzimidazolium moieties (Figure 8 A). These dihedral angles are reduced markedly, to 24.4° (19.35°, 24.17°, 29.64°), when one additional electron is added to the trication, in its LUMO, reducing the molecular charge from +3 to +2. The optimized geometry of the **8** ^2+^ species is considerably more planar and has a larger resemblance to the optimized molecular geometry obtained for the trication‐graphene unit cell (Figure 8 B) than that of the trication itself (Figure 8 A). This indicates that binding of the trication to the graphene surface may be accompanied by transfer of some electron density from graphene to the trication.

To identify the effect of electron transfer, calculations were performed with different overall charges (neutral, **8^1+^**, **8^2+^** and **8^3+^**) of the unit cell (Table 2). For the overall **8^3+^** charged unit cell the calculations show that graphene donates substantial electron density, approximately corresponding to half an electron, into the LUMO of the tricationic molecule. The LUMO orbital of the trication is mainly localized on the central benzene ring and it is bonding between this ring and (two of the three) the peripheral benzimidazolium moieties. From a stabilizing point of view the effect of this partial electron transfer is twofold. First, the increased electron density at the central benzene ring decreases the coulombic repulsion between the cationic benzimidazolium moieties via a screening effect. Second, the bonding traits of the LUMO results in a planarization of the overall molecular structure when electron density is fed into the orbital. The later contributes favorably to the bonding interaction by allowing for a shorter bonding distance and an increase in the surface area exposed to graphene by the trication. The denser deposition of molecules in the physical samples compared to the unit cell explored in the calculations may disturb the system causing some molecules on the graphene surface to possess more **8^2+^** character and others to be more like **8^3+^**. This could explain why the adsorbed molecules display broad/overlapping nitrogen signals in XPS. Another reason could be that the strong binding in combination with the propeller shaped molecular structure results in two chemically distinctly different sets of nitrogen: the set of three nitrogen atoms close to the graphene surface and the set of those on the molecular face opposite to the graphene surface. All six nitrogen atoms in the free cation are chemically equivalent and should, as observed for the pristine silicon/silicate substrate, only give one peak in XPS. The single main peak may in fact indicate that the interaction between the trication and the silicon/silicate surface is so weak that it does not significantly disturb the molecular properties.

**Table 2:** Supercell starting charge and overall electron distribution of molecule **8^3+^** (without counterions), when non‐covalently bound to graphene.

Supercell starting charge	Graphene final charge [e]	Molecule final charge [e]	Charge transferred from graphene [e]
**8^0^**	−1.93	+1.72	≈−1.18
**8^1+^**	−0.94	+2.02	≈−1.02
**8^2+^**	−0.32	+2.21	≈−0.74
**8^3+^**	+0.61	+2.48	≈−0.55

**Figure 9:**
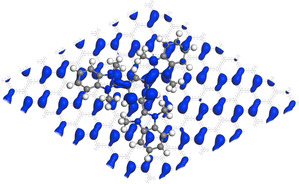
Partial charge density of HOMO for optimized geometry of **8^3+^** charged graphene‐trication unit cell. It shows a large charge density localized on the central benzene ring of the **8^3+^** trication (without counterions). The charge density localized on the molecule corresponds to the LUMO orbital for the isolated trication.

The same LUMO orbital, responsible for the stabilization effect discussed for the **8^3+^** charged unit cell, is the one accepting a substantial amount of the charge density of the additional electron in the **8^2+^** charged unit cell (it can be pictured as a unit cell assembled from a trication and a monoanionic supercell of graphene, Figure 9, Figure S43). The computed electron densities show that the charge of graphene is only slightly negative and that the charge of the molecule is close to **8^2+^**. Further decrease of overall charge of the unit cell in the calculations shows consistently a charge density on the molecule approximately corresponding to a **8^2+^** species. The charge density corresponding to the additional electron instead builds up on graphene (Table 2). Charge transfer from graphene to the trication and the formation of a highly delocalized positive charge could rationalize the decrease in the amount of counterions observed in XPS. To better understand the nature of the charge transfer we have, moreover, calculated the electron affinity of tricationic **8^3+^** (−4.1 eV) and the ionization potential of graphene (exptl. 7.20–7.30 eV). The mismatch between the two values justifies the partial transfer of charge (not a full electron) between graphene and trication in case of the **8^3+^** unit cell.

**Figure 10:**
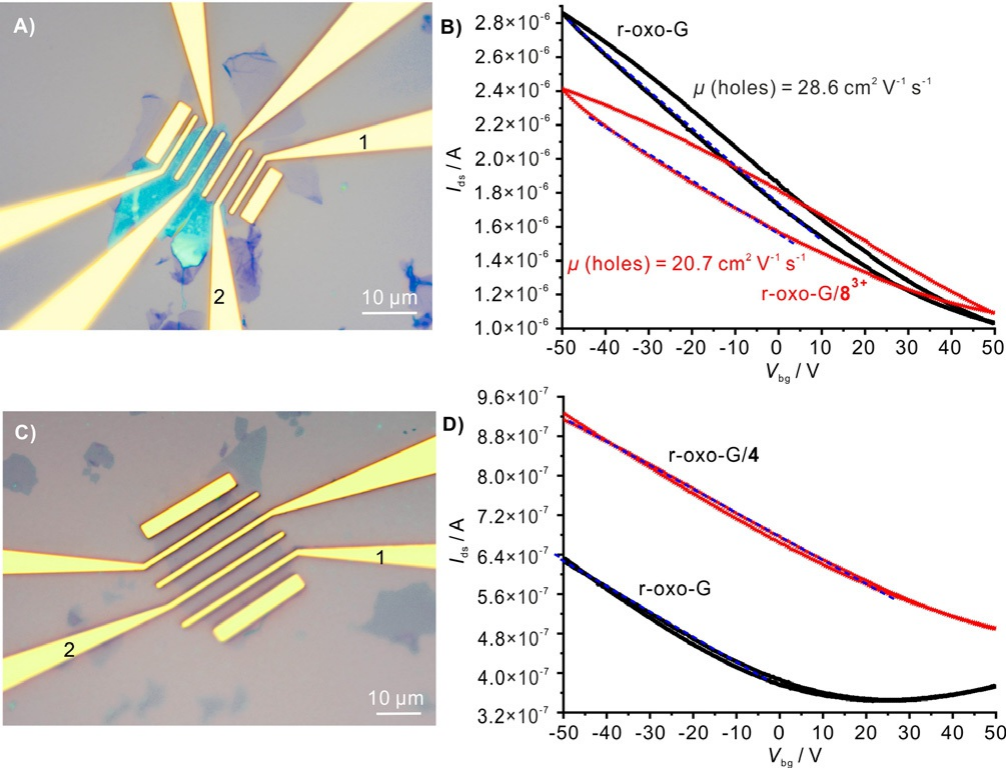
Transport measurements of monolayer r‐oxo‐G‐based FET. A) Optical image of the fabricated graphene device. A channel width of 20 μm and length of 3 μm between electrode 1 and 2. B) Transfer characteristics of the monolayer graphene before and after doping with **8^3+^**. Transport measurements of monolayer r‐oxo‐G‐based FET. *V*_ds_=10 mV. C) Optical image of the fabricated r‐oxo‐G device. A channel width of 10 μm and length of 4.5 μm between electrode 1 and 2. D) Transfer characteristics of the monolayer r‐oxo‐G before and after doping with **4**. *V*_ds_=10 mV.

To better investigate the p‐doping effect of **8^3+^** on graphene, r‐oxo‐G was used to fabricate a monolayer field‐effect transistor device (FET) (Figure 10 A). In recent investigations, we evaluated the transport properties of reduced oxo‐G with different densities of defects and the effect of the substrate.^21^, ^35^ Here, the FET device was tested before and after non‐covalent modification. The electrical transport measurements were performed in a two‐probe configuration at ambient conditions. The Si/300 nm SiO_2_ substrate was used as back gate and source‐drain voltage (*V_ds_*) was constant at 10 mV. Transfer curves (*I_ds_*‐*V_ds_*) with unipolar character are shown in Figure 10 B. The hysteresis effect of the monolayer r‐oxo‐G on SiO_2_ is observed after sweeping continuously from −50 to 50 V and then back to −50 V. From the dashed lines (on black curve in Figure 10 B), a hole mobility (*μ_h_*) of 28.6 cm^2^ V^−1s−1^ is obtained at ambient conditions, calculated by Equation (1):

(1) $$\mu =\left(L/W\right)\times \left({1/(C}_{ox}V_{ds}\right))\times \left(dI_{ds}/dV_{bg}\right)$$

in which *C_ox_*=1.15×10^−8^ F/cm^−2^. After doping r‐oxo‐G with **8^3+^** *I_ds_*‐*V_ds_* curves still keep the unipolar character, but these curves lean to a higher *p*‐doped direction. The hole mobility decreased to 20.7 cm^2^ V^−1s−1^. In addition, we found that the hysteresis effect increased indicating that more carriers were trapped between r‐oxo‐G and the SiO_2_ interfaces. The resistance of monolayer r‐oxo‐G device (with length 3 μm) before and after doping varies only slightly from 6.0 to 5.8 kΩ with a channel width of 20 μm and length of 3 μm. To investigate the influence of the hysteresis on the carrier mobilities deeper, we calculated the carrier mobilities at different slopes for r‐oxo‐G and r‐oxo‐G/**8^3+^**, respectively, (Figure S48).

We note that the reference experiment using the uncharged compound **4** for non‐covalent functionalization of r‐oxo‐G also results in *p*‐doping of the underlying graphene (Figure 10 C). Transfer curves (*I_ds_*‐*V_ds_*) in Figure 10 D show a Dirac point at 20 V, which indicates that r‐oxo‐G itself possessing p‐doping feature, which can stem from both oxygen and water present under ambient conditions. We also observe a negligible hysteresis effects from the measurements with **4**, compared with the measurements from **8^3+^**. After doping with **4**, the transfer curves become unipolar, suggesting a stronger *p*‐doping character of r‐oxo‐G. Hole mobility (*μ_h_*) of the r‐oxo‐G before and after doping decreases from 20.4 cm^2^ V^−1^ s^−1^ to 18.9 cm^2^ V^−1^ s^−1^. The corresponding resistance of the r‐oxo‐G before and after doping decreases from 27.2 to 18.9 kΩ at *V_ds_*=10 mV, *V_bg_*=0 V with a channel width of 10 μm and length of 4.5 μm. Comparing both FET devices with each other, a stronger decrease in hole mobility can be observed for **8^3+^**. This can be explained by a stronger interaction between **8^3+^** and r‐oxo‐G compared with **4** and r‐oxo‐G, which leads simultaneously to a stronger p‐doping effect on the underlying graphene for **8^3+^**.

## Conclusions

Through a concise synthetic strategy, we synthesized a series of cationic *N*‐hetero‐π‐systems, which were used to non‐covalently functionalize graphene forming graphene/molecular hybrid structures. The cationic molecules form a stable molecular layer and interact strongly with graphene. This results in a reversible one‐electron transfer between the molecules and the underlying graphene, which leads to p‐doping of the graphene. The functionalization of graphene and the one‐electron transfer was studied by AFM, XPS, ToF‐SIMS and Raman spectroscopy. The performed measurements did not show any influence of the various counterions. DFT‐calculations confirmed the observed effects from the performed experiments. With the reported wet‐chemical method of graphene functionalization, we were able to fabricate monolayer graphene FET devices. Comparing both FET devices with each other, a stronger decrease in hole mobility can be observed for the tricationic molecules **8^3+^** compared to the neutral precursor **4**. This can be explained by a stronger interaction between **8^3+^** and r‐oxo‐G compared with **4** and r‐oxo‐G, which leads simultaneously to a stronger *p*‐doping effect on the underlying graphene for trication **8^3+^**.

Both the novel series of compounds as well as the described method help to understand the non‐covalent functionalization of graphene in more detail. In the future, novel sensors based on FET devices can be fabricated for selective sensing, if the analyte interacts with the cationic molecules adsorbed to the surface.

## Conflict of interest

The authors declare no conflict of interest.

## Supporting information

As a service to our authors and readers, this journal provides supporting information supplied by the authors. Such materials are peer reviewed and may be re‐organized for online delivery, but are not copy‐edited or typeset. Technical support issues arising from supporting information (other than missing files) should be addressed to the authors.

Supplementary
