# Supplementary material for: Evidence for Electron Transfer between Graphene and Non‐Covalently Bound π‐Systems
Source: Chemistry. 2020 Apr 17;26(29):6694–702. doi: 10.1002/chem.202000488 (PMC7317416; doi:10.1002/chem.202000488)
Supplement: Supplementary file 1 — Supplementary [file CHEM-26-6694-s001.pdf]

Chemistry–A European Journal

Supporting Information

Evidence for Electron Transfer between Graphene and Non-Covalently Bound π-Systems

Steffen M. Brülls,[a] Valentina Cantatore,[a] Zhenping Wang,[b] Pui Lam Tam,[c] Per Malmberg,[a] Jessica Stubbe,[d] Biprajit Sarkar,[d, e] Itai Panas,[a] Jerker Mårtensson,[a] and Siegfried Eigler*[b]

Table of Contents

TABLE OF CONTENTS 1
EXPERIMENTAL PROCEDURES 2
SYNTHETIC PROCEDURES 4
2,2',2''-((BENZENE-1,3,5-TRIYL)TRIS(1,3-DIMETHYL-1*H*-BENZO[*D*]IMIDAZOL-3-IUM)TRIFLATE (8^{3+}) 4
2,2',2''-(BENZENE-1,3,5-TRIYL)TRIS(3-ETHYL-1-METHYL-1*H*-BENZO[*D*]IMIDAZOL-3-IUM)TRIFLATE(11^{3+}) 4
2,2',2''-(BENZENE-1,3,5-TRIYL)TRIS(1-METHYL-3-OCTYL-1*H*-BENZO[*D*]IMIDAZOL-3-IUM)TRIFLATE(13^{3+}) 5
2,2',2''-((BENZENE-1,3,5-TRIYL)TRIS(1,3-DIMETHYL-1*H*-BENZO[*D*]IMIDAZOL-3-IUM) TETRAFLUOROBORATE (9^{3+}) 5
2,2',2''-(BENZENE-1,3,5-TRIYL)TRIS(3-ETHYL-1-METHYL-1*H*-BENZO[*D*]IMIDAZOL-3-IUM) TETRAFLUOROBORATE (12^{3+}) 6
2,2',2''-((BENZENE-1,3,5-TRIYL)TRIS(1,3-DIMETHYL-1*H*-BENZO[*D*]IMIDAZOL-3-IUM) IODIDE (7^{3+}) 6
SPECTROSCOPIC CHARACTERIZATION OF COMPOUNDS 7
RESULTS AND DISCUSSION 17
HR-MS SPECTRA: 17
RAMAN SPECTROSCOPY: 19
X-RAY PHOTOELECTRON SPECTROSCOPY 21
IMAGING TIME-OF-FLIGHT SECONDARY ION MASS SPECTROMETRY 25
ATOMIC FORCE MICROSCOPY 28
DENSITY FUNCTIONAL THEORY CALCULATIONS 28
CYCLIC VOLTAMMETRY CHARACTERIZATION OF 9^{3+}: 30
CYCLIC VOLTAMMETRY CHARACTERIZATION OF 12^{3+}: 31
IN-SITU SPECTROSCOPY OF 12^{3+}: 32
TRANSPORT MEASUREMENTS 33
REFERENCES 33

Experimental Procedures

All starting materials were purchased as reagent grade from *Sigma- Aldrich*. The reagents were used as received. Dry acetonitrile was purchased from *Acros* and stored under N_2-atmosphere. Octyltriflate[1] and 1,3,5-tris(benzimidazolyl)benzene **3**[2] and **4**[2] were prepared according to literature procedures. Thin layer chromatography (TLC) was performed on plates from *Merck* (silica gel 60, F254). Substances were visualized under UV light (wavelength λ = 254 nm). Solvents and R_f values are stated in the experimental part. Column chromatography was performed on silica gel from *Merck* (35-70 micron). Some purifications were performed *via* automated column chromatography on a Biotage IsoleraTM Spektra One flash chromatography system usingBiotage SNAP-50 g KP-sil columns. The CVD graphene was purchased from *Graphenea*. Its quality was checked by analysis of the G and 2D peaks *via* Raman spectroscopy. Air-sensitive reactions were carried out in flame-dried glassware and under an inert N_2-atmosphere using Schlenk techniques. Melting points were determined on a Büchi B-545 melting-point apparatus in open capillaries and are reported uncorrected. “Decomp” refers to decomposition.

2D material sample preparation

The sample preparation of non-covalent functionalized graphene for HR-MS, Raman spectroscopy, XPS, AFM and electrical transport measurements was always the same. A silicon wafer with deposited graphene, either CVD graphene or r-oxo-G was incubated in a 12 mM methanol solution of the respective tricationic molecule for 2 hours at 4 °C. After incubation, the functionalized graphene wafer was removed from the incubation solution and rinsed with methanol to remove excess of the tricationic molecules. XPS samples and Raman spectroscopy samples of neat molecules/adsorbates without graphene were prepared by drop-casting 3 drops of a 12 mM methanol solution of the respective tricationic molecule onto a silicon wafer and allow the solvent to evaporate.

Nuclear magnetic resonance spectroscopy

^{1}H NMR, ^{13}C NMR and ^{19}F spectra were recorded on a Agilent 400 spectrometer (400.1 MHz for ^{1}H, 100.6 MHz for ^{13}C and 376.3 MHz for ^{19}F). Chemical shifts (δ) are reported in ppm and were referenced to the residual solvent signal as an internal reference (DMSO-d_6: 2.50 ppm for ^{1}H, 39.52 for ^{13}C; ACN-d_3: 1.94 ppm for ^{1}H, 1.32, 118.26 for ^{13}C; CD_2Cl_2: 5.32 ppm for ^{1}H, 53.84 for ^{13}C). Coupling constants (*J*) are given in Hertz (Hz) and the apparent resonance multiplicity is reported as singlet (s), doublet (d), triplet (t), quartet (q) or multiplet (m).

High resolution mass spectroscopy

High resolution mass spectroscopy data was obtained on an Agilent 6520 QTOF LC/MS coupled with an Agilent 1290 Infinity LC system. The signal of the molecular ion $[M]^+$ is reported in m/z units. The submitted samples (1 mg) were diluted to ca. 10 µg/ml in acetonitrile. The sample was analyzed using an Agilent 1290 infinity LC system equipped with autosampler tandem. HRMS spectra were recorded with a 0.3 ml/min flow rate using an isocratic method (50% MPA/50% MPB). Mobile Phase A (MPA): Water with 0.04% formic acid. Mobile Phase B (MPB): MeOH with 0.04% Formic acid. All samples were initially analyzed using an ESI source in positive mode (scan range 100-1200 m/z). Samples were also analyzed in negative mode to detect anionic molecules and fragments (scan range 50-1200 m/z).

Raman spectroscopy

Raman spectroscopy data was obtained on a WITec alpha300 R instrument using an excitation wavelength of 532 nm. The laser intensity was kept at 1.7 mW for all performed measurements unless stated otherwise. The integration time was 0.1 s. Single spectra were four times accumulated. For most measurements a grating of 600 g/mm was chosen and the spectral center was set to 1900 cm^{-1}. For a grating of 1800 g/mm, the spectral center was set to 1350 cm^{-1}. The sample was placed on a motorized x,y table and focused before each measurement. For larger areas it was ensured that the focus of the excitation laser was constant. The data analysis was performed with the Project Manager software from WITec and visualized with OriginPro 9. The G and the 2D peaks were fitted by one Lorentzian function each. The graphene samples were analyzed via Raman spectroscopy before and after non-covalent modification.

X-ray photoelectron spectroscopy

X-ray photoelectron spectroscopy (XPS) experiment was conducted in a PHI5000 VersaProbe III Scanning XPS Microprobe system. The X-ray source was a monochromated Al anode (E = 1486.6 eV) with the beam diameter 100 µm (Energy resolution: 0.646 eV). Dual charge compensation was conducted by using argon ion gun (+ve) and electron neutralizer (-ve) for non-conductive materials. The survey scan was performed in the range between 0 and 1250 eV (step size: 1.0 eV) for compositional analysis. The high resolution narrow scan for the selected regions was performed with the step size of 0.1 eV for chemical state analysis. The energy scale calibration (ISO 15472) was performed by aligning the core level peaks of $Au4f_{7/2}$, $Ag3d_{5/2}$ and $Cu2p_{3/2}$ at 83.96 eV, 368.21 eV and 932.62 eV, respectively. The sample was adhered on the platen with double-sided tape, and the surface contaminant was removed by blowing with dry N_2 gas. The analyzing chamber was kept under UHV condition with pressure lower than 3.0 x 10^{-6} Pa. The data analysis was performed with MultiPak software (Ver. 9.7.0.1). To ease data analysis, three energy states, including C1s at 284.6 eV[3] for the sp^2-hybridised carbon in graphene (i.e. C=C bond), Si2p at 103.6 – 104.0 eV and O1s at 532.6 – 533.0 eV for the native SiO_2 layer on silicon wafer, are used accordingly as the reference positions in this study.The used CVD graphene just showed the elemental signals for carbon, oxygen and silicon (from the wafer) in the measurements (see **Figure S27**).

Imaging time-of-Flight Secondary Ion Mass Spectrometry

ToF-SIMS data were obtained with a TOF.SIMS 5 instrument (ION-TOF GmbH, Münster, Germany). The instrument is equipped with a 25 keV $Bi3^+$ cluster ion gun as the primary ion source and a 10 keV C_{60}^+ ion source for sputtering and etching. The samples were analyzed using a pulsed primary ion beam ($Bi3^{++}$, 0.14 pA at 50 keV) at a field of view of 500 µm × 500 µm. All spectra were acquired and processed with the Surface Lab software (version 6.4, ION-TOF GmbH). Low-energy electrons were used for charge compensation during analysis.

Oxo-Graphene and electrical measurements

The electrical transport measurements were performed in a two-probe configuration at ambient conditions. Oxo-Graphene flakes were deposited onto the Si/SiO_2 (300 nm) substrate by Langmuir–Blodgett technique (LB, Kibron µtrough). Reduction was performed by vapor of hydriodic acid and trifluoroacetic acid (1/1 mixture by volume) at 80 °C. Reduced oxo- Graphene was cleaned with doubly distilled water (Carl Roth). Patterning of the electrode structure was achieved by standard electron beam lithography processing (Raith PIONEER TWO). The 5/70 nm Cr/Au electrodes were deposited by thermal evaporation (Kurt J. Lesker NANO 36). All electrical measurements were carried out at ambient conditions using a two-probe station with micromanipulated probes and two source-measurement units (Keithley 2450). Oxo-graphene and r-oxo-G were synthesized according to a previously published literature.[4]

Atomic Force microscopy

AFM characterization was performed using a JPK Nanowizzard 4 atomic force microscope in tapping mode at room temperature.

Density functional theory calculations

For all the results presented in this work, a 9 × 9 graphene supercell (162 atoms) with the molecule M adsorbed on top was employed. All the results were obtained using the CASTEP program package within the Material Studio 2017 framework in conjunction with the PBE functional including the semi-empirical dispersion-corrected DFT approach (DFT-D2)[5] of Grimme. Core electrons were described by on-the-fly ultrasoft pseudopotentials and 517 eV cut-off energy. Atomic positions were optimized with the BFGS algorithm using delocalized internal coordinates. The evaluation of the partial atomic charges on the graphene and on the molecule was made based on Mulliken population analysis provided by the DFT calculations.

CV/UV-Vis-NIR spectroelectrochemistry:

Cyclic voltammetry was carried out in 0.1 M Bu_4NPF_6 solution using a three-electrode configuration (glassy carbon working electrode, Pt counter electrode, Ag wire as pseudoreference) and PAR VersaSTAT 4 potentiostat. The sample concentration was 10^{-4} M. The ferrocene/ferrocenium (Fc/Fc^+) couple served as internal reference. Spectroelectrochemical measurements were carried out in an optically transparent thin-layer electrochemical (OTTLE)[6] cell (CaF_2 windows) with a platinum-mesh working electrode, a platinum-mesh counter electrode, and a silver-foil pseudoreference electrode. Anhydrous and degassed ACN with 0.1 M NBu_4PF_6 as electrolyte was used as the solvent.

Synthetic procedures

2,2',2''-((benzene-1,3,5-triyl)tris(1,3-dimethyl-1*H*-benzo[*d*]imidazol-3-ium)triflate (8^{3+})

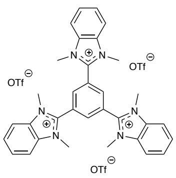

Method A: 1,3,5-tris(1-methyl-1*H*-benzo[*d*]imidazol-2-yl)benzene[2] (100 mg, 0.21 mmol, 1.0 equiv.) was suspended in 10 ml dry acetonitrile under a dry nitrogen atmosphere. Methyl trifluoromethansulfonate (114 mg, 0.70 mmol, 3.3 equiv.) was added and the reaction mixture was stirred at room temperature for 24 h. Acetonitrile was removed under reduced pressure. The product was precipitated from methanol/diethyl ether to give 175 mg of product in 87% yield as white solid. Method B: 1,3,5-tris(1*H*-benzo[*d*]imidazol-2-yl)benzene[1] (50.0 mg, 0.12 mmol, 1.0 equiv.) and potassium carbonate (50.0 mg, 0.36 mmol, 3 equiv.) were suspended in 10 ml dry acetonitrile and stirred at room temperature for 30 minutes under a dry nitrogen atmosphere. Methyl trifluoromethansulfonate (0.16 ml, 1.40 mmol, 12.0 equiv.) was added by a syringe and the reaction mixture was stirred for 90 minutes at 50 °C. After allowed to cool down to room temperature acetonitrile was removed under reduced pressure. The crude product was redissolved in methanol and precipitated by slow addition of diethyl ether to give 120 mg of product in 60% yield as a white solid.

^{1}H NMR (400 MHz, acetonitrile-d$_3$) δ 4.07 (s, 18H, N-C*H*$_3$), 7.81–7.88 (m, 6H, Ar*H*), 7.98–8.05 (m, 6H, Ar*H*), 8.58 (s, 3H, Ar*H*);
^{13}C NMR (101 MHz, acetonitrile-d$_3$) δ 34.02, 114.25, 118.32, 125.22, 128.52, 133.26, 138.59, 148.12;
^{19}F NMR (101 MHz, acetonitrile-d$_3$) δ -79.41.
HRMS (ESI); Exact mass calculated for cation $(C_{33}H_{33}N_6)^{3+}$ [M+3]: 171.0917, Observed Mass: 171.0924.
HRMS (ESI); Exact mass calculated for anion $(C_2H_3F_3O_3S)^-$ [M-]: 148.9520, Observed Mass: 148.9524.
mp: 290 °C.

2,2',2''-(benzene-1,3,5-triyl)tris(3-ethyl-1-methyl-1*H*-benzo[*d*]imidazol-3-ium)triflate(11^{3+})

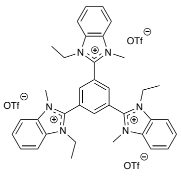

1,3,5-tris(1-methyl-1*H*-benzo[*d*]imidazol-2-yl)benzene[2] (100 mg, 0.21 mmol, 1. 0equiv.)) was suspended in 10 ml dry acetonitrile under a dry nitrogen atmosphere. Ethyl trifluoromethansulfonate (125 mg, 0.70 mmol, 3.3 equiv.) was added by a syringe and the reaction mixture was stirred at room temperature for 24 h. Acetonitrile removed under reduced pressure. The crude product was redissolved in methanol and precipitated by slow addition of diethyl ether to give 129 mg of product in 59% yield as a white solid.

^{1}H NMR (400 MHz, acetonitrile-d$_3$) δ 1.55 (t, 9H, *J* = 7.3 Hz, 9H, C*H*$_3$) 4.02 (s, 9H, N-C*H*$_3$), 4.52 (q, 6H, *J* = 7.3 Hz, 6H, N-C*H*$_2$-CH$_3$), 7.83–7.88 (m, 6H, Ar*H*), 7.98–8.11 (m, 6H, Ar*H*), 8.50 (s, 3H, Ar*H*);
^{13}C NMR (101 MHz, acetonitrile-d$_3$) δ 14.21, 33.03, 42.31, 113.59, 117.32, 124.75, 127.72, 131.08, 132.59, 137.08, 146.51;
^{19}F NMR (101 MHz, acetonitrile-d$_3$) δ -79.59.
HRMS (ESI); Exact mass calculated for cation $(C_{36}H_{39}N_6)^{3+}$ [M+3]: 185.1073, Observed Mass: 185.1078.
HRMS (ESI); Exact mass calculated for anion $(C_2H_3F_3O_3S)^-$ [M-]: 148.9520, Observed Mass: 148.9525.
mp: 237 °C.

2,2',2''-(benzene-1,3,5-triyl)tris(1-methyl-3-octyl-1*H*-benzo[*d*]imidazol-3-ium)triflate(13^{3+})

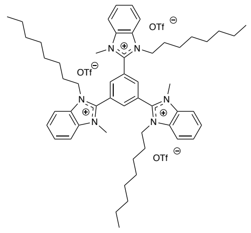

1,3,5-tris(1-methyl-1*H*-benzo[*d*]imidazol-2-yl)benzene[2] (50.0 mg, 0.10 mmol, 1.0 equiv.) was suspended in 10 ml dry acetonitrile under a dry nitrogen atmosphere. Octyltrifluoromethan sulfonate[1] (91.0 mg, 0.33 mmol, 3.3 equiv.) was added by a syringe and the reaction mixture was stirred at room temperature for 24 h. Acetonitrile was removed under reduced pressure. The crude product was redissolved in methanol and precipitated by slow addition of diethyl ether to give 38 mg of product in 30% yield as a white solid.

^{1}H NMR (400 MHz, acetonitrile-d_3) δ 0.88 (t, *J* = 7.0 Hz, 9H, C*H*$_3$), 1.19–1.39 (m, 36H, (C*H*$_2$)$_6$), 3.99 (s, 9H, N-C*H*$_3$), 4.41 (t, *J* = 7.7 Hz, 6H, N-C*H*$_2$), 7.82–7.89 (m, 6H, Ar*H*), 7.98–8.08 (m, 6H, Ar*H*), 8.49 (s, 3H, Ar*H*);
^{13}C NMR (101 MHz, acetonitrile-d_3) δ 13.37, 22.35, 26,42, 28.94, 29.25, 31.52, 33.12, 46.93, 113.75, 117.01, 124.83, 127.88, 131.39, 132.44, 137.17, 146.29;
^{19}F NMR (101 MHz, acetonitrile-d_3) δ -79.39.
HRMS (ESI); Exact mass calculated for cation $(C_{54}H_{75}N_6)^{3+}$ [M+3]: 269.2012, Observed Mass: 269.2018.
HRMS (ESI); Exact mass calculated for anion $(C_2H_3F_3O_3S)^-$ [M-]: 148.9520, Observed: 148.9526.

2,2',2''-((benzene-1,3,5-triyl)tris(1,3-dimethyl-1*H*-benzo[*d*]imidazol-3-ium) tetrafluoroborate (9^{3+})

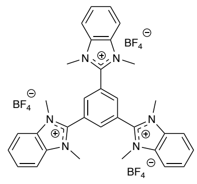

1,3,5-tris(1-methyl-1*H*-benzo[*d*]imidazol-2-yl)benzene[2] (50.0 mg, 0.10 mmol, 1.0 equiv.) was suspended in 10 ml dry acetonitrile under a dry nitrogen atmosphere. Trimethyloxonium tetrafluoroborate (49.0 mg, 0.33 mmol, 3.3 equiv.) was added and the reaction mixture was stirred at room temperature for 24 h. Acetonitrile was removed under reduced pressure. The crude product was redissolved in methanol and precipitated by slow addition of diethyl ether to give 72 mg of product in 88% yield as a white solid.

^{1}H NMR (400 MHz, acetonitrile-d_3) δ 4.07 (s, 18H, N-C*H*$_3$), 7.84–7.88 (m, 6H, Ar*H*), 7.99–8.04 (m, 6H, Ar*H*), 8.49 (s, 3H, Ar*H*);
^{13}C NMR (101 MHz, acetonitrile-d_3) δ 33.06, 113.39, 117.32, 124.25, 127.74, 132.25, 137.53, 147.11;
^{19}F NMR (101 MHz, acetonitrile-d_3) δ -151.78.
HRMS (ESI); Exact mass calculated for cation $(C_{33}H_{33}N_6)^{3+}$ [M+3]: 171.0917, Observed Mass: 171.0924.
HRMS (ESI); Exact mass calculated for anion $(BF_4)^-$ [M-]: 87.0035, Observed Mass: 87.0039.
mp: > 350 °C.

2,2',2''-(benzene-1,3,5-triyl)tris(3-ethyl-1-methyl-1*H*-benzo[*d*]imidazol-3-ium) tetrafluoroborate (12^{3+})

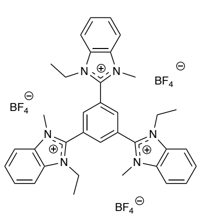

1,3,5-tris(1-methyl-1*H*-benzo[*d*]imidazol-2-yl)benzene[2] (50.0 mg, 0.10 mmol, 1.0 equiv.) was suspended in 10 ml dry acetonitrile under a dry nitrogen atmosphere. Then triethyloxonium tetrafluoroborate (60.0 mg, 0.33 mmol, 3.3 equiv.) was added and the mixture was stirred at room temperature for 24 h. Acetonitrile was removed under reduced pressure. The crude product was redissolved in methanol and precipitated by slow addition of diethyl ether to give 49 mg of product in 58% yield as a white solid.

^{1}H NMR (400 MHz, acetonitrile-d$_3$) δ 1.51 (t, *J* = 7.3 Hz, 9H, C*H*$_3$), 3.98 (s, 9H, N-C*H*$_3$), 4.48 (q, *J* = 7.2 Hz, 6H, N-C*H*$_2$), 7.79–7.84 (m, 6H, Ar*H*), 7.97–8.03 (m, 6H, Ar*H*), 8.44 (s, 3H, Ar*H*);
^{13}C NMR (101 MHz, acetonitrile-d$_3$) δ 15.19, 33.94, 43.25, 114.58, 125.75, 128.76, 132.05, 133.56, 137.98, 147.47;
^{19}F NMR (101 MHz, acetonitrile-d$_3$) δ -151.83.
HRMS (ESI); Exact mass calculated for cation ($C_{35}H_{36}N_6^{3+}$)$^{3+}$ [M+3]: 185.1073, Observed Mass: 185.1081.
HRMS (ESI); Exact mass calculated for anion (BF_4)$^-$ [M-]: 87.0035, Observed Mass: 87.0039.
mp: 200 °C.

2,2',2''-((benzene-1,3,5-triyl)tris(1,3-dimethyl-1*H*-benzo[*d*]imidazol-3-ium) iodide (7^{3+})

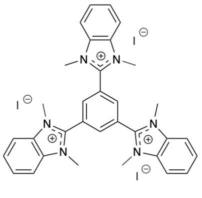

1,3,5-tris(1*H*-benzo[*d*]imidazol-2-yl)benzene[2] (50.0 mg, 0.12 mmol, 1.0 equiv.) and potassium carbonate (50.0 mg, 0.36 mmol, 3.0 equiv.) were suspended in 10 ml dry acetonitrile and stirred at room temperature for 30 minutes under a dry nitrogen atmosphere. Then methyl iodide (0.45 ml, 1.40 mmol, 12.0 equiv.) was added by a syringe and the reaction mixture was stirred for 90 minutes at 50 °C. After cooling down to room temperature acetonitrile was removed under reduced pressure. The crude product was redissolved in methanol and precipitated by slow addition of diethyl ether to give 34 mg of product in 30% yield as a white solid.

^{1}H NMR (400 MHz, DMSO-d$_6$) δ 4.08 (s, 18H, N-C*H*$_3$), 7.81–7.86 (m, 6H, Ar*H*), 8.18–8.23 (m, 6H, Ar*H*), 8.83 (s, 3H, Ar*H*). mp: 250 °C (decomp.).
^{13}C NMR (101 MHz, DMSO-d$_6$) δ 33.35, 113.64, 122.98, 127.18, 131.88, 138.01, 147.78;
mp: 250 °C Decomp.

(2-(3,5-bis(3-ethyl-1-methyl-1*H*-3λ^4-benzo[*d*]imidazol-1-ium-2-yl)phenyl)-1-ethyl-1*H*-3λ^4-benzo[*d*]imidazol-1-ium-3-yl)methanide (10^{3+})

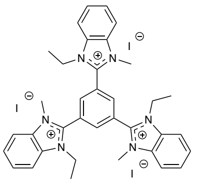

1,3,5-tris(1-methyl-1*H*-benzo[*d*]imidazol-2-yl)benzene[2] (50.0 mg, 0.10 mmol, 1.0 equiv.) was suspended in 10 ml dry acetonitrile under a dry nitrogen atmosphere. Then ethyl iodide (51.5 mg, 0.33 mmol, 3.3 equiv.) was added and the mixture was stirred at room temperature for 24 h. Acetonitrile was removed under reduced pressure. The crude product was redissolved in methanol and precipitated by slow addition of diethyl ether to give 49 mg of product in 58% yield as a white solid.
^{1}H NMR (400 MHz, DMSO-d$_6$) δ 1.44 (t, *J* = 7.3 Hz, 9H, C*H*$_3$), 4.04 (s, 9H, N-C*H*$_3$), 4.53 (q, *J* = 7.2 Hz, 6H, N-C*H*$_2$), 7.83–7.85 (m, 6H, Ar*H*), 8.22–8.27 (m, 6H, Ar*H*), 8.84 (s, 3H, Ar*H*); **^{13}C NMR** (101 MHz, DMSO-d$_6$) δ 14.52, 33.05, 113.77, 3.83, 123.63, 127.10, 127.16, 130.80, 137.31, 147.34, 165.54; **mp**: 250 °C Decomp.

Spectroscopic Characterization of Compounds

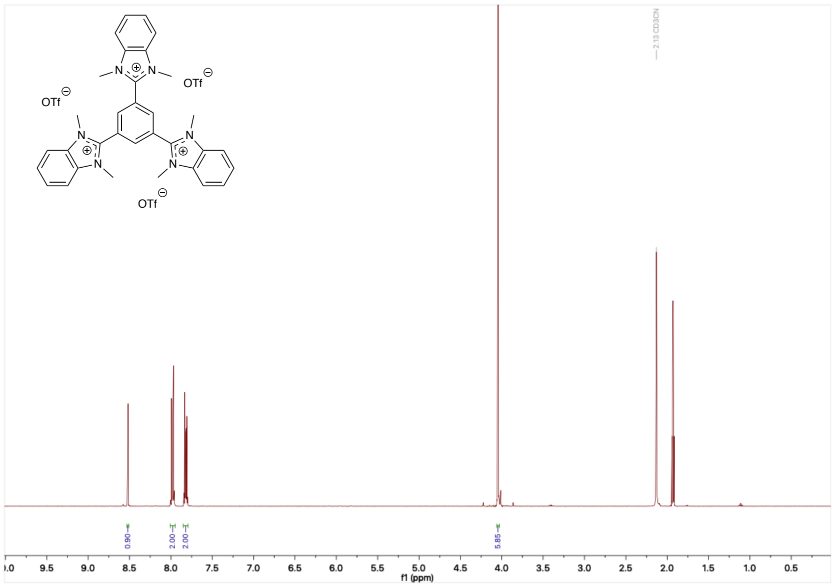

Figure S1. ^{1}H NMR in ACN-d_3 of compound **8**$^{3+}$

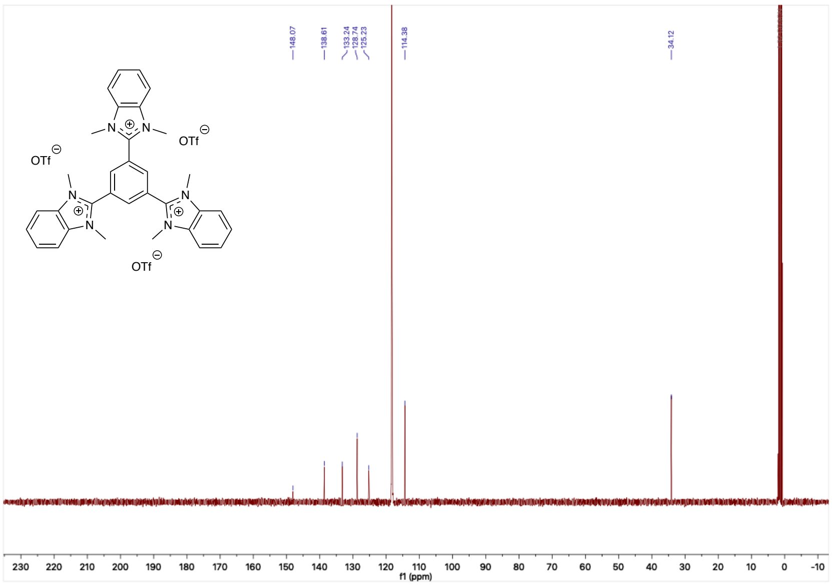

Figure S2. ^{13}C NMR in ACN-d_3 of compound **8**$^{3+}$**.**

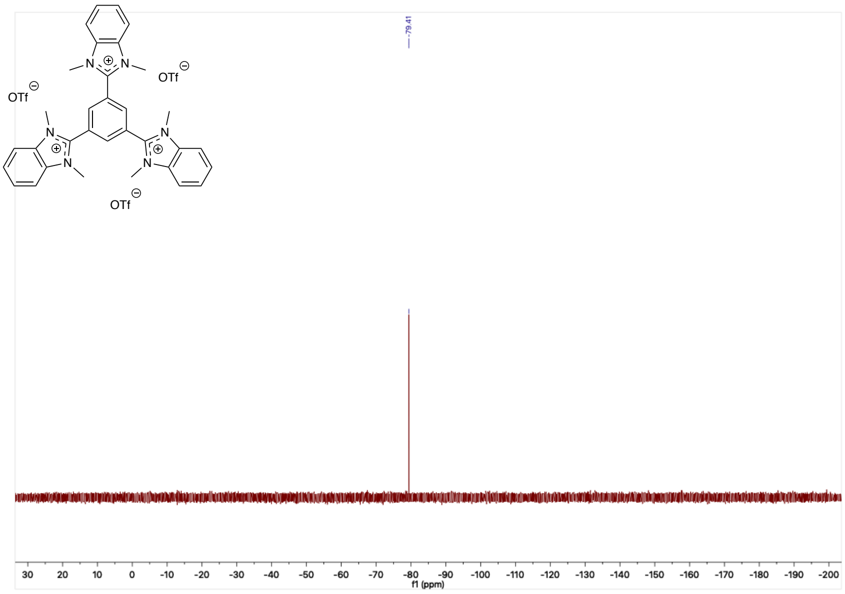

Figure S3. ^{19}F NMR in ACN-d$_3$ of compound **8^{3+}**.

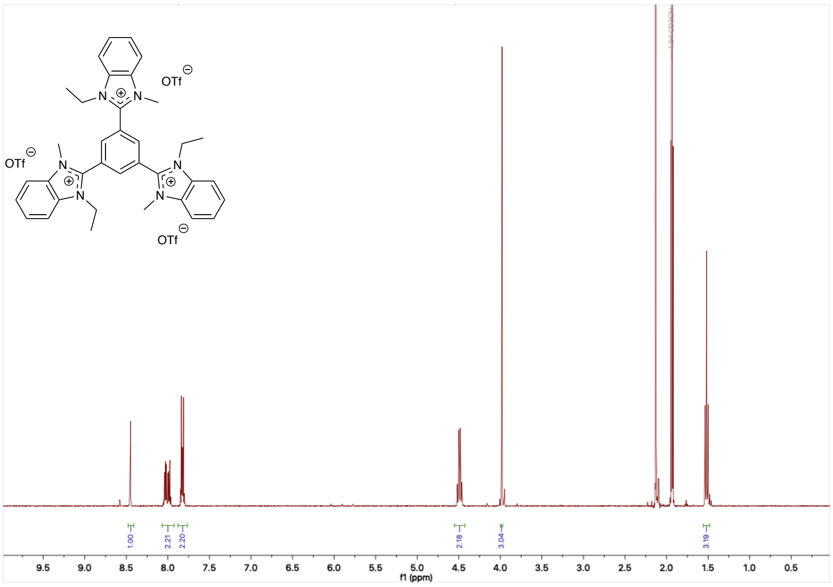

Figure S4. ^{1}H NMR in ACN-d$_3$ of compound **11^{3+}.**

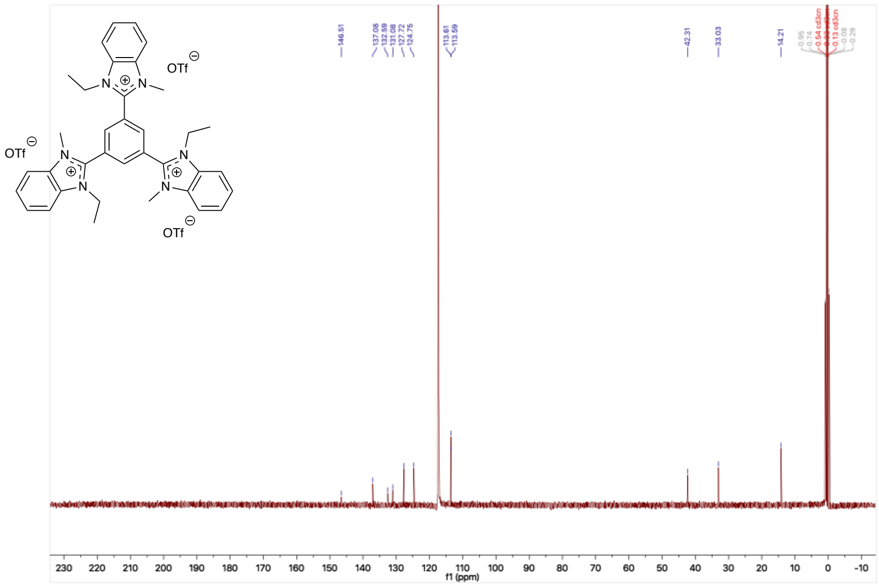

Figure S5. ^{13}C NMR in ACN-d_3 of compound **11^{3+}**.

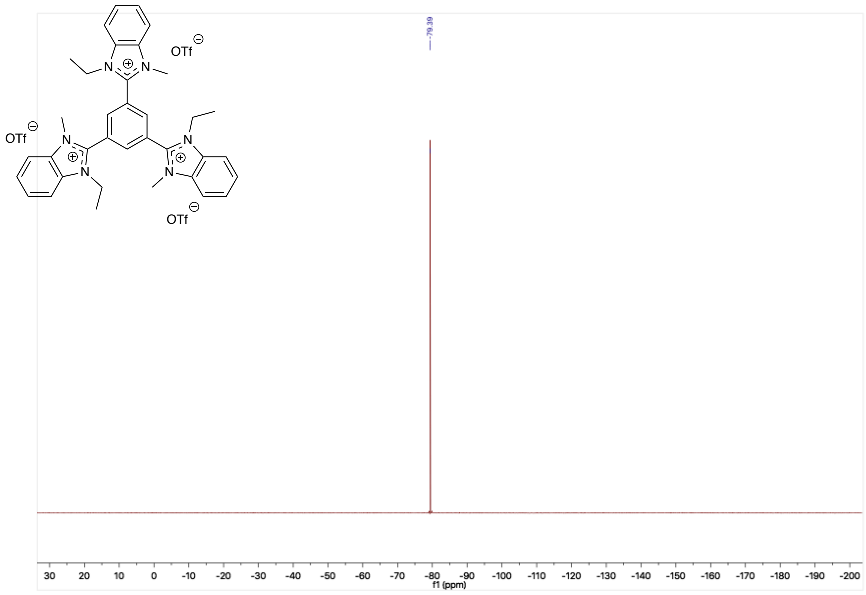

Figure S6. ^{19}F NMR in ACN-d_3 of compound **11^{3+}**.

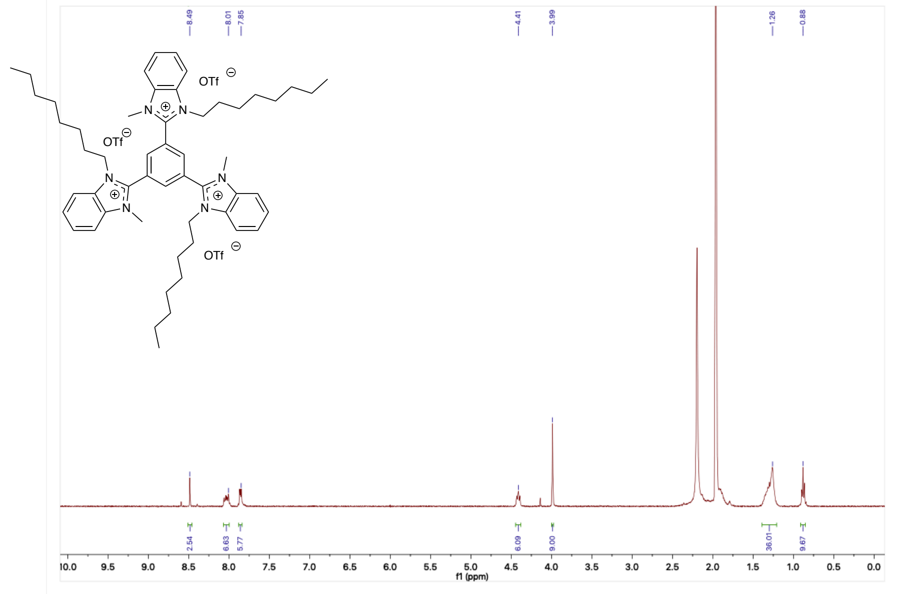

Figure S7. ^{1}H NMR in ACN-d_3 of compound **13^{3+}**.

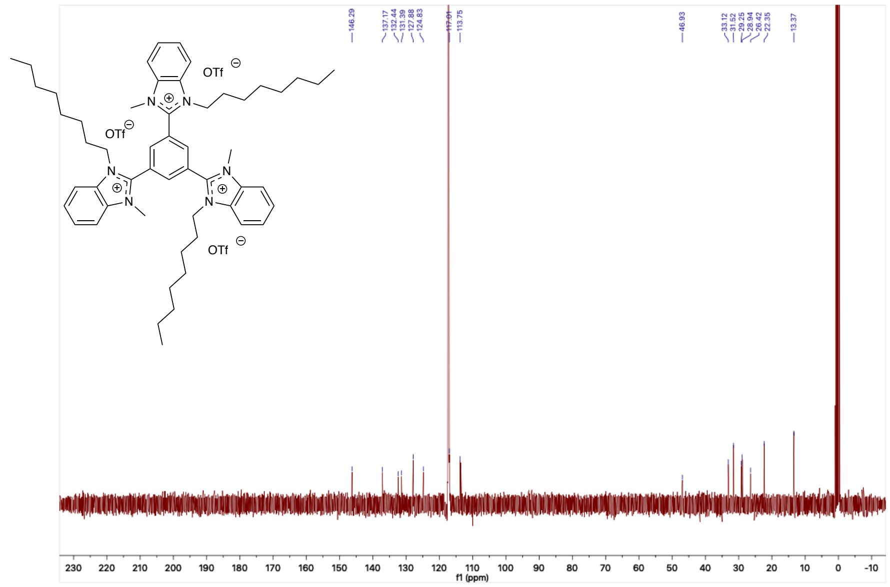

Figure S8. ^{13}C NMR in ACN-d_3 of compound **13^{3+}**.

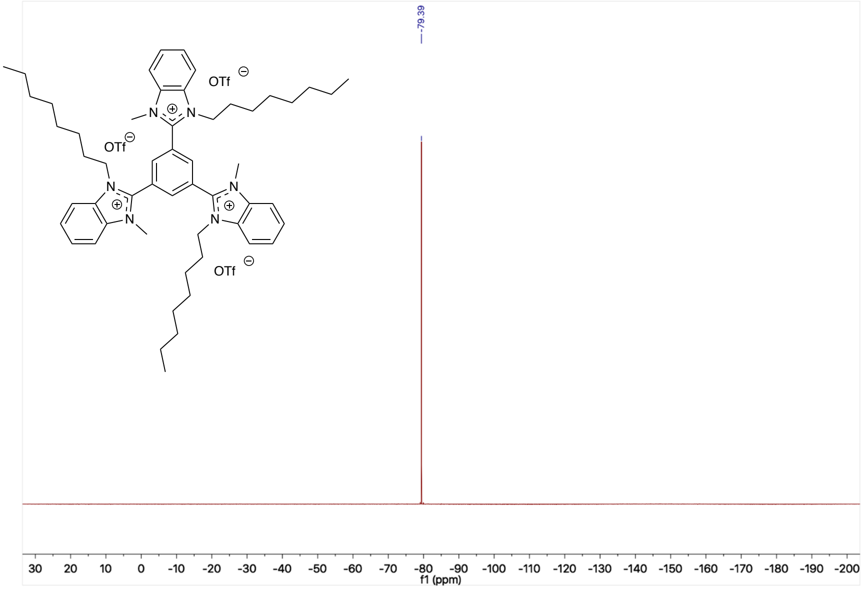

Figure S9. ^{19}F NMR in ACN-d$_3$ of compound **13^{3+}**.

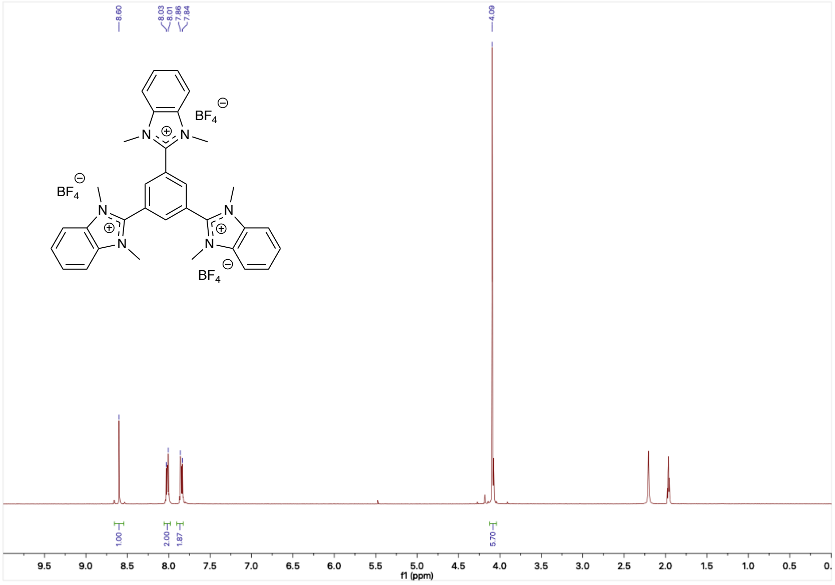

Figure S10. ^{1}H NMR in ACN-d$_3$ of compound **9^{3+}**.

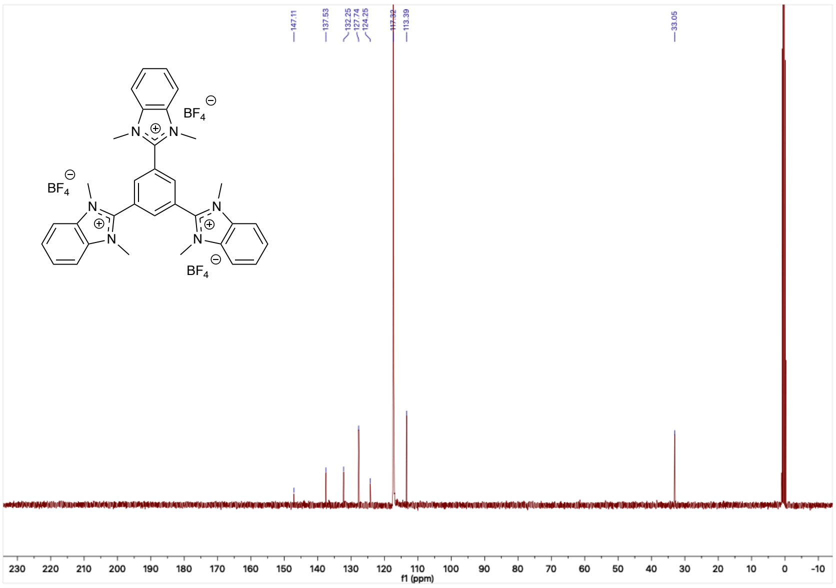

Figure S11. ^{13}C NMR in ACN-d3 of compound **9**$^{3+}$.

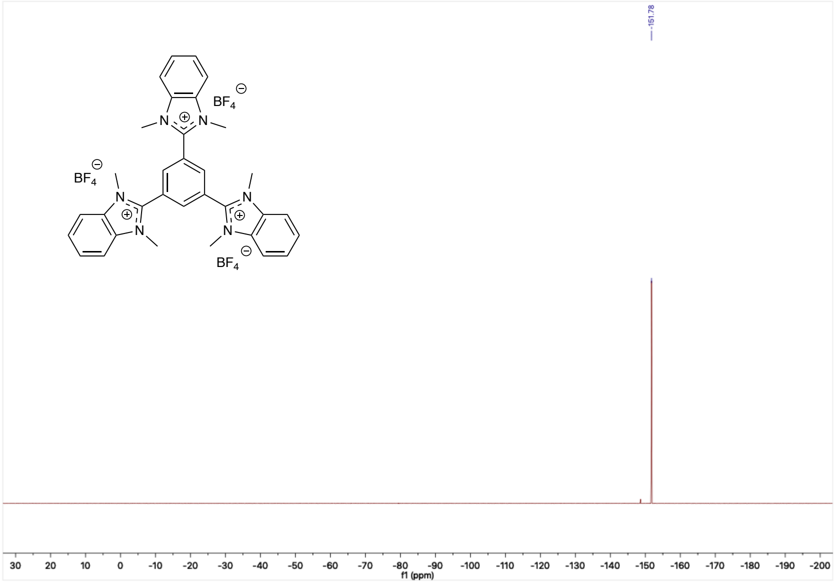

Figure S12. ^{19}F NMR in ACN-d3 of compound **9**$^{3+}$.

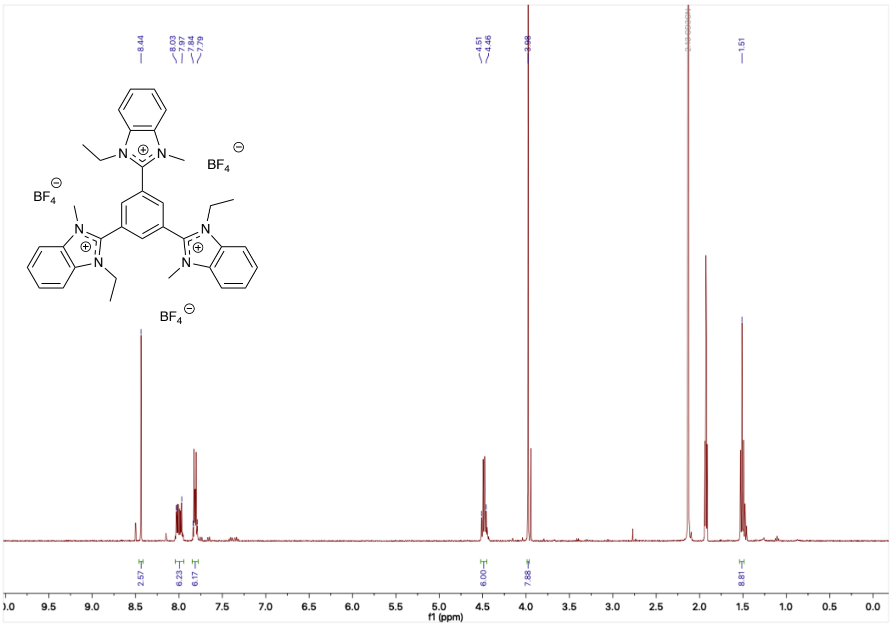

Figure S13. ^{1}H NMR in ACN-d$_3$ of compound **12^{3+}**.

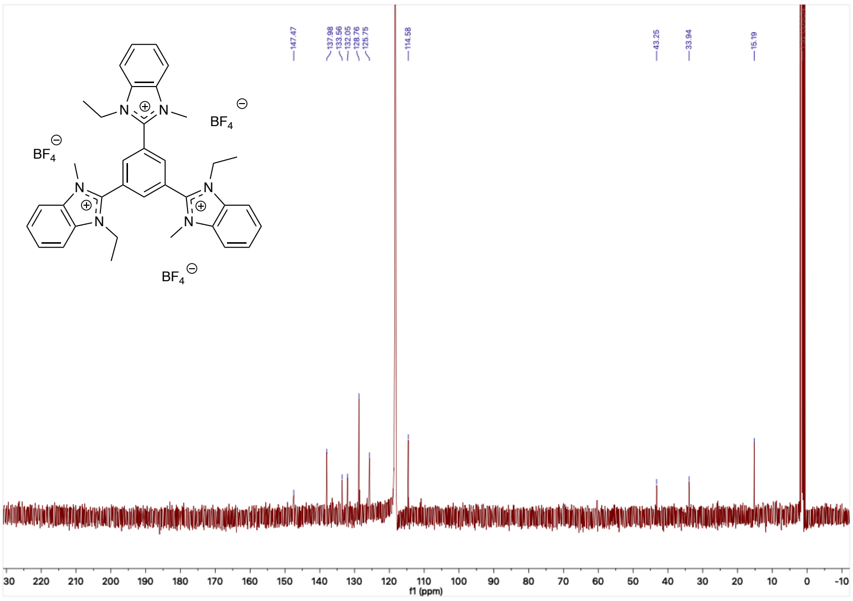

Figure S14. ^{13}C NMR in ACN-d$_3$ of compound **12^{3+}**.

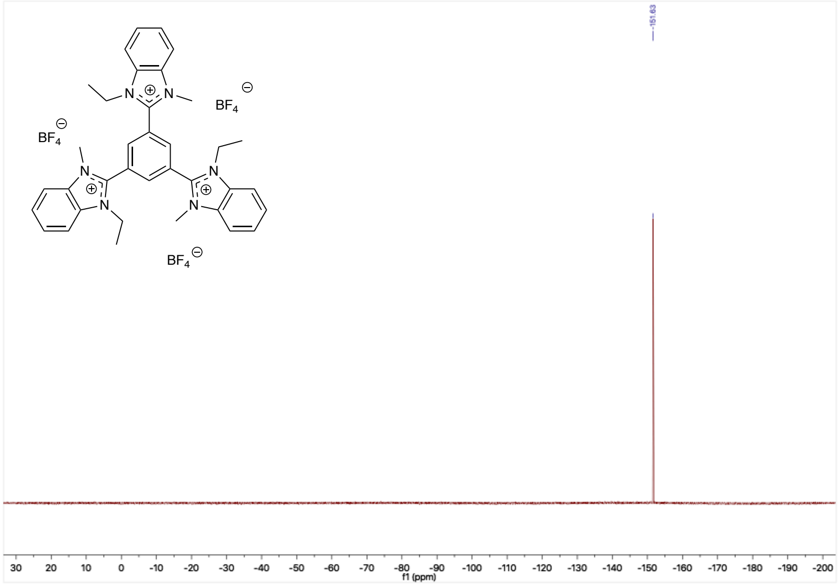

Figure S15. ^{19}F NMR in ACN-d$_3$ of compound **12^{3+}**.

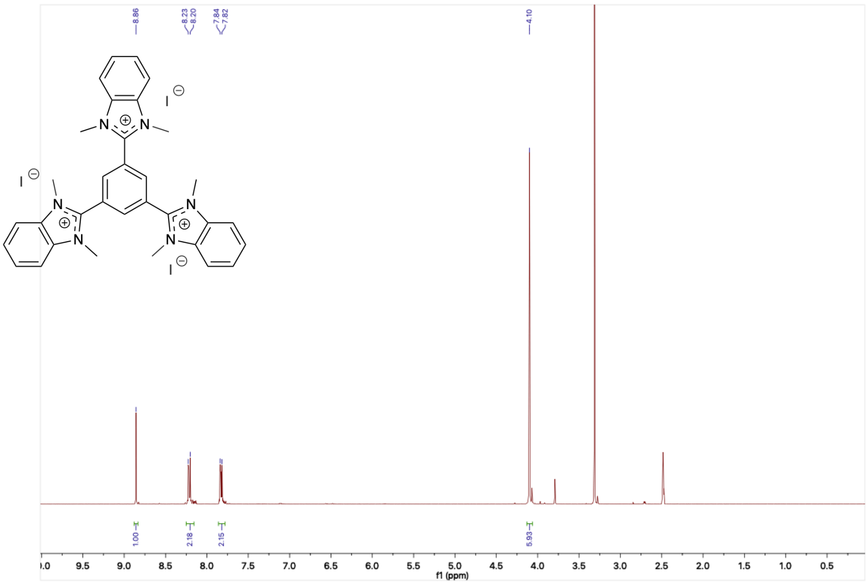

Figure S16. ^{1}H NMR in DMSO-d$_6$ of compound **7^{3+}**.

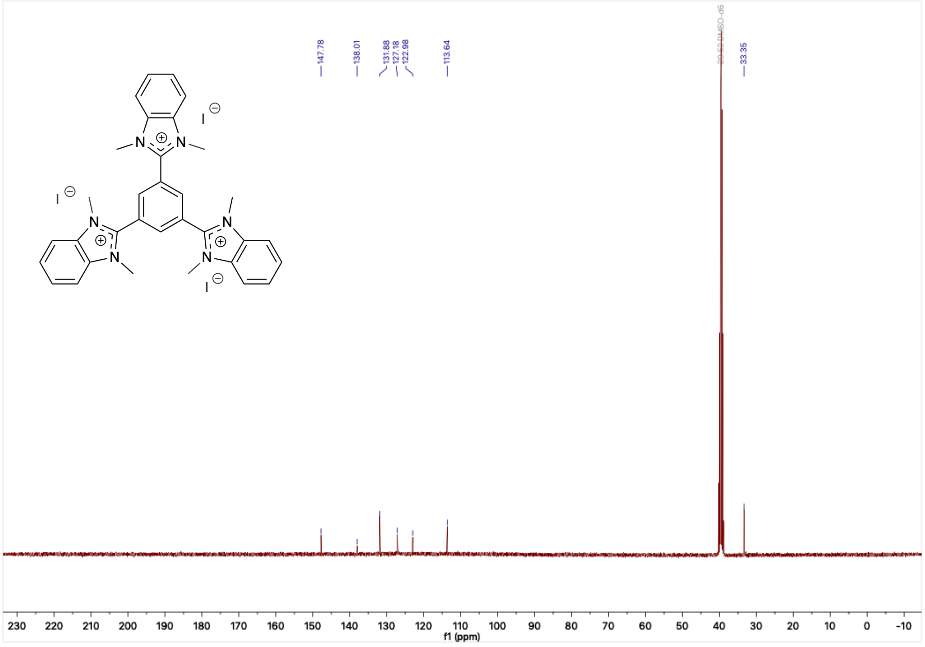

Figure S17. ^{13}C NMR in DMSO-$d_6$of compound **7^{3+}**.

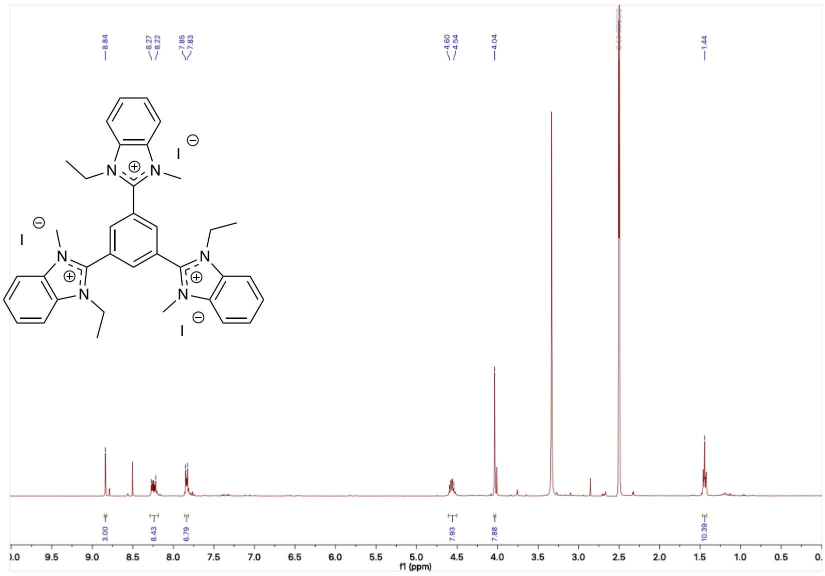

Figure S18. ^{1}H NMR in DMSO-d_6 of compound **10^{3+}**.

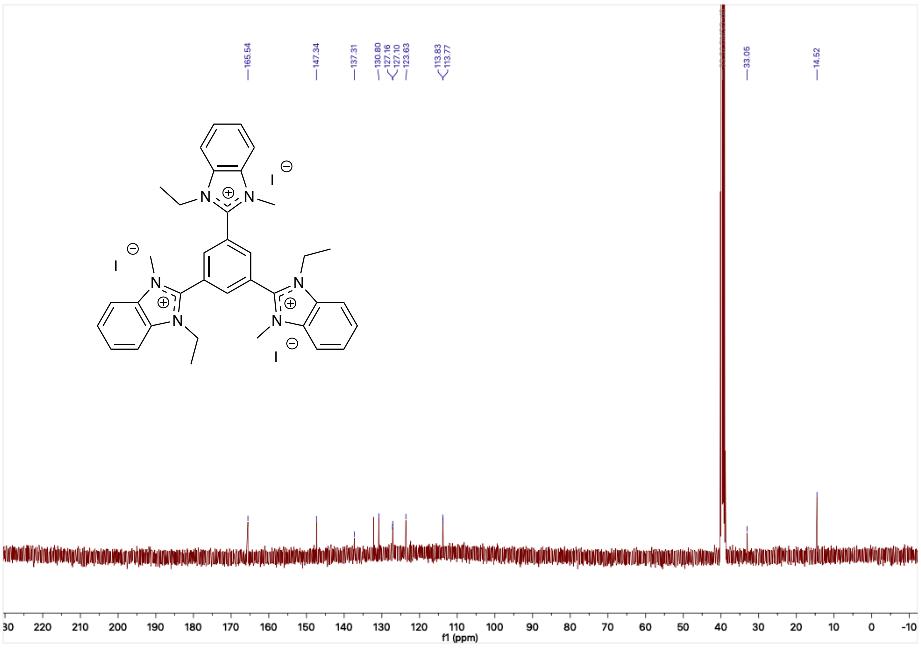

Figure S19. ^{13}C NMR in DMSO-d_6 of compound **10^{3+}**.

HR-MS spectra:

This paragraph shows the HR-MS spectra from **8**$^{3+}$, **9**$^{3+}$, **11**$^{3+}$, **13**$^{3+}$. Compounds **7**$^{3+}$, **10**$^{3+}$ and **12**$^{3+}$ could be not protonated with ESI.

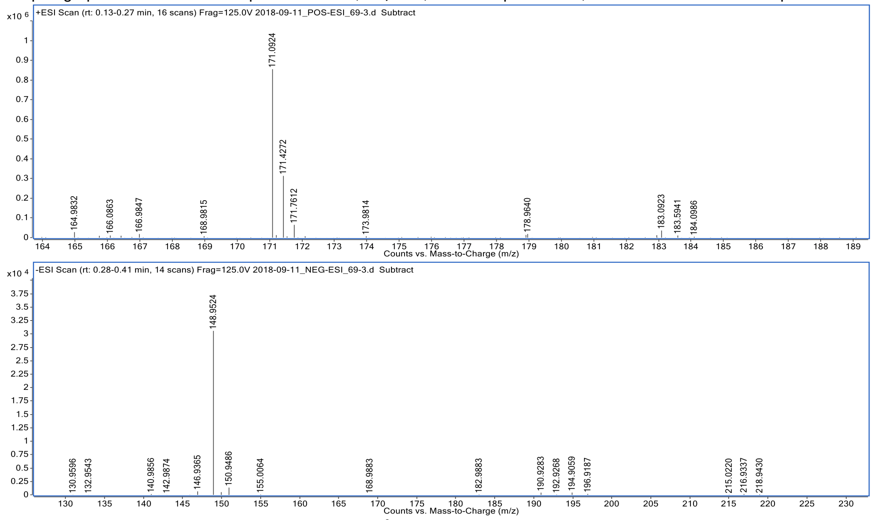

Figure S20. HR-MS spectra of compound **8**$^{3+}$. Positive ion mode on the top and negative ion mode on the bottom.

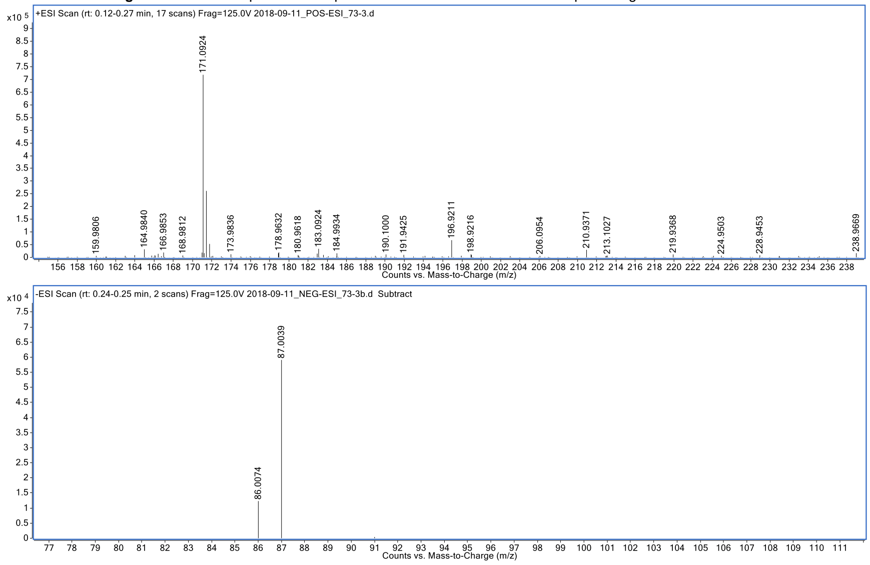

Figure S21. HR-MS spectra of compound **9**$^{3+}$. Positive ion mode on the top and negative ion mode on the bottom.

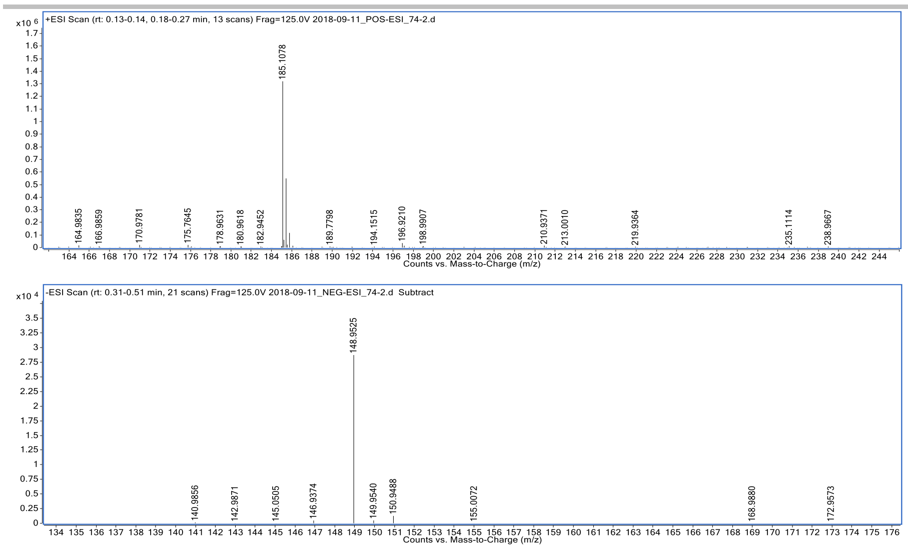

Figure S22. HR-MS spectra of compound **11**$^{3+}$. Positive ion mode on the top and negative ion mode on the bottom.

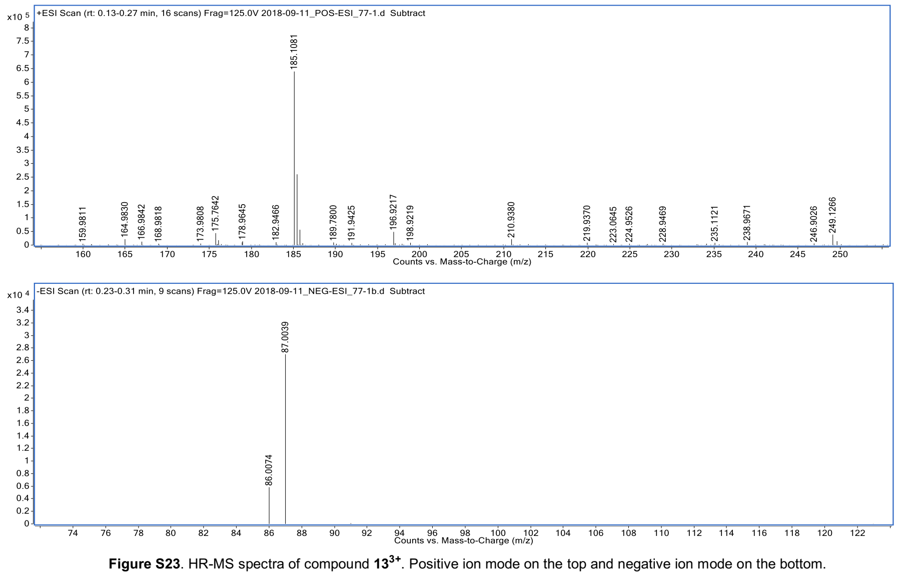

Figure S23. HR-MS spectra of compound **13**$^{3+}$. Positive ion mode on the top and negative ion mode on the bottom.

Raman spectroscopy:

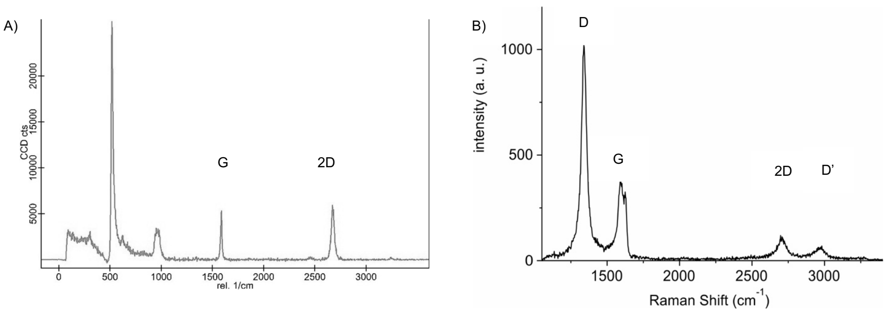

Figure S24. Raman spectrum of A) CVD graphene sample (unfunctionalized) showing the characteristic G and 2D bands of graphene and B) r-oxo-graphene sample showing the characteristic D (defect), G, 2D and D' bands of graphene.

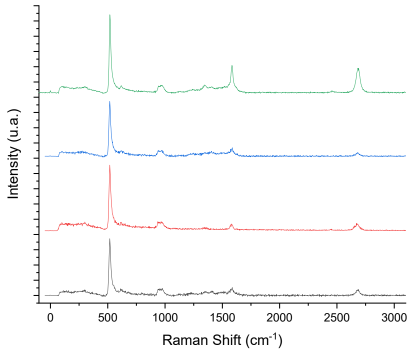

Figure S25. Raman spectra of non-covalently functionalized CVD graphene. Green curve: functionalized with **12**$^{3+}$, blue curve: functionalized with **11**$^{3+}$, red curve: functionalized with **7**$^{3+}$ derivative, black curve: functionalized with **5**$^{3+}$.

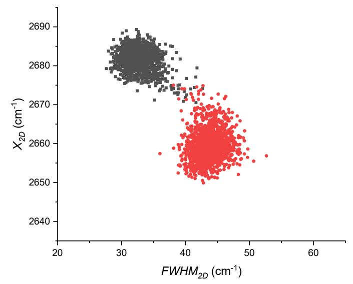

Figure S26. Scatter plot of the 2D peak of CVD graphene (black) and with molecule **8**$^{3+}$ functionalized CVD graphene (red).

A significant change between the CVD graphene and the non-covalently functionalized graphene can be observed by analyzing the 2D peak for both samples. The CVD graphene give a 2D peak at 2682 cm^{-1} (median value, **Figure S26**, right side, black). The position of the 2D peak in the non-covalently functionalized graphene is at 2659 cm^{-1} (median value, Figure S26, right side, red). This corresponds to a shift of the 2D peak of 23 cm^{-1}. The FWHM Γ_{2D} of the 2D peak of the graphene sample is 32.6 cm^{-1} (median value, **Figure S26**, right side in black) and broadens to 43.7 cm^{-1} (median value **Figure S26**, right side in red) for the non-covalently functionalized graphene sample. That leads to a shift of 11.2 cm^{-1} of Γ_{2D}.

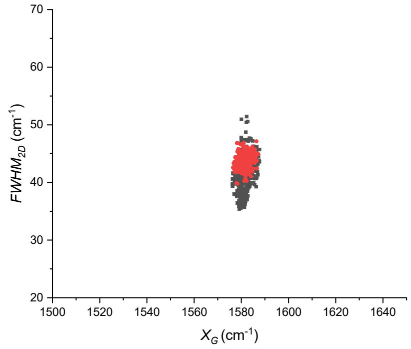

Figure S27. Scatter plot of CVD graphene before (black) and after (red) 2 hours incubation in methanol. The used CVD graphene sample has a X_G of 1581.4 cm^{-1}(median value). After incubation in methanol the position of the G band does not change (median value after incubation, 1581.5 cm^{-1}). The position of the full width at half maximum (FWHM) Γ_G of the CVD sample is at 41.2 cm^{-1} (median value) and changes slightly to 43.8 cm^{-1}(median value) after the 2 hours of incubation in methanol. Surface scan area: 5 µm x 5 µm.

X-ray photoelectron spectroscopy

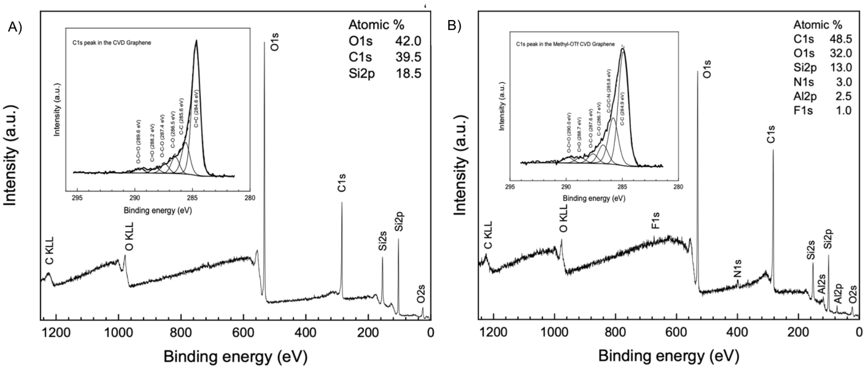

Figure S28. XPS survey spectrum of A) CVD graphene and the corresponding spectrum of C1s peak and B) molecule **8**$^{3+}$ on CVD graphene and the corresponding spectra of the C1s peaks.

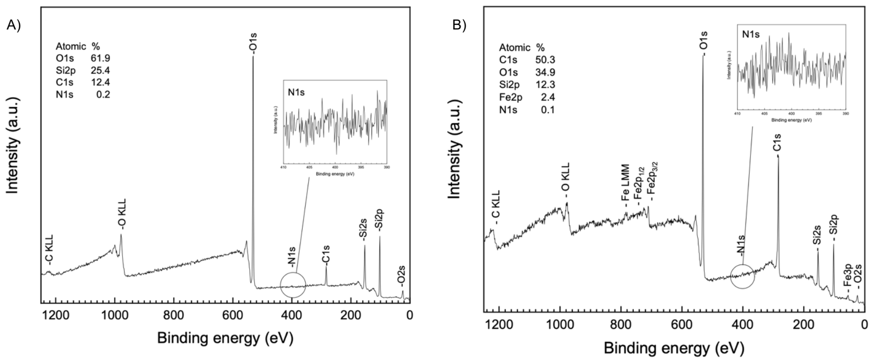

Figure S29. XPS survey spectrum of the A) uncoated silicon and B) the CVD graphene and the corresponding spectra of N1s peak.

Feldfunktion geändert

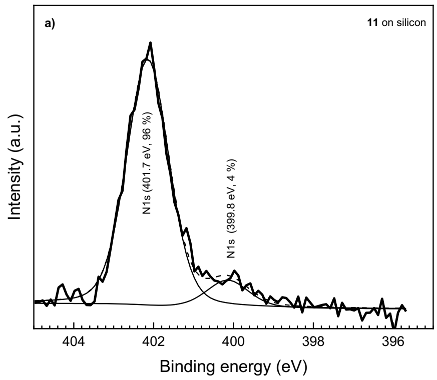

Feldfunktion geändert

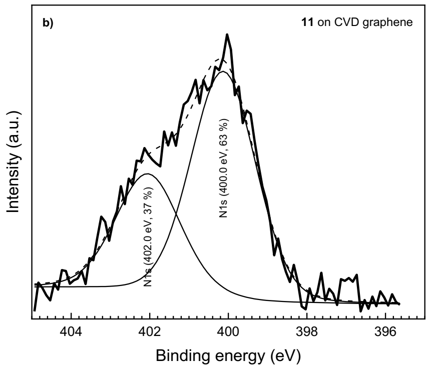

Figure S30. High resolution scan at the N1s region for a) **11**$^{3+}$ on silicon and b) **11**$^{3+}$ on CVD graphene.

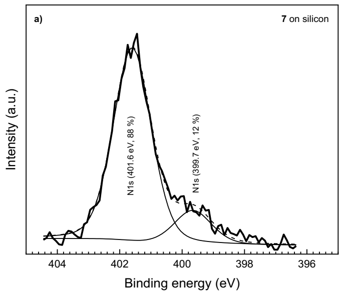

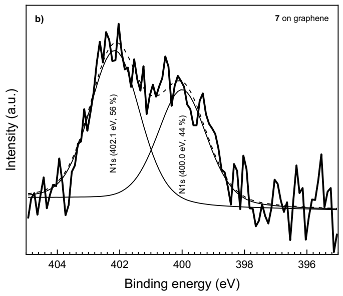

Figure S31. High resolution scan at the N1s region for a) $\mathbf{7}^{3+}$ on silicon and b) $\mathbf{7}^{3+}$ on CVD graphene.

Feldfunktion geändert

Feldfunktion geändert

Imaging Time-of-Flight Secondary Ion Mass Spectrometry

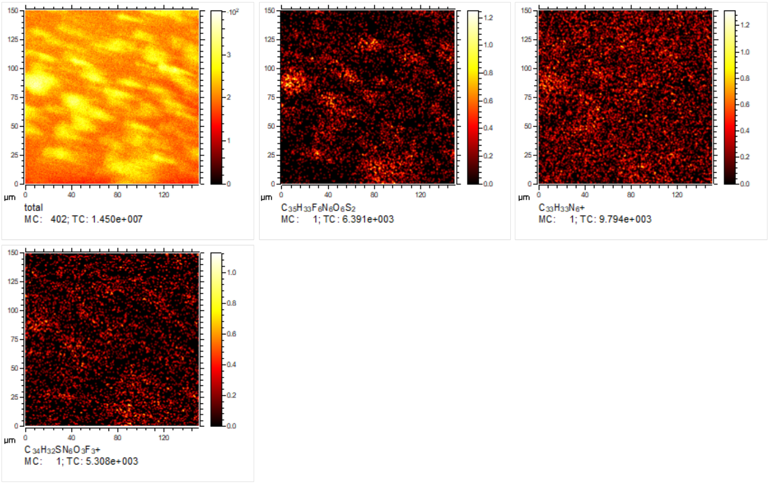

Figure S32. ToF-SIMS images in positive ion mode showing a) total amount of found m/z signals, b) m/z = 787.2 c) m/z = 513.28 and d) m/z = 662.73. Surface scan area: 150 µm x 150 µm.

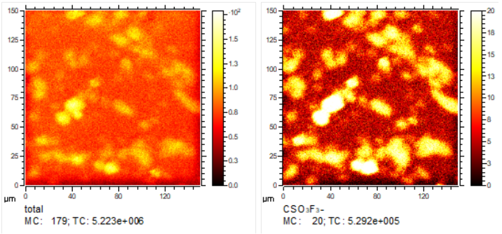

Figure S33. ToF-SIMS images in negative ion mode showing a) total amount of found m/z signals and b) m/z = 148.95. Surface scan area: 150 µm x 150 µm.

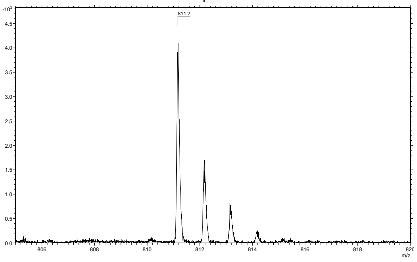

Figure S34. ToF-SIMS spectrum in positive ion mode showing m/z = 811.2.

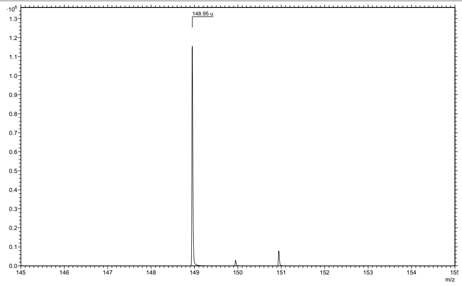

Figure S35. ToF-SIMS spectrum in negative ion mode showing m/z = 148.95.

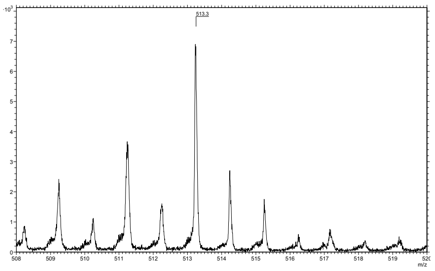

Figure S36. ToF-SIMS spectrum in positive ion mode showing m/z = 512.3.

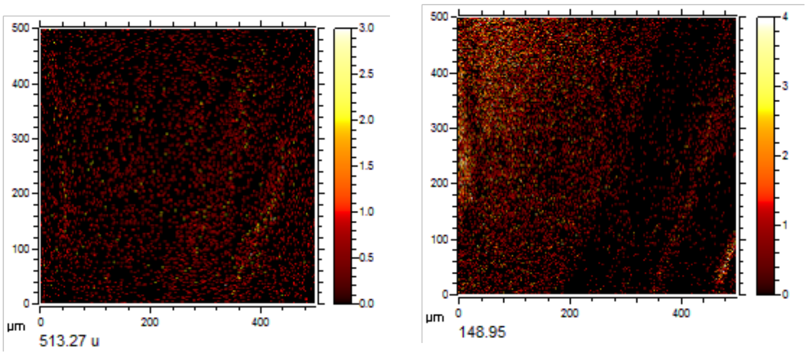

Figure S37. ToF-SIMS images of CVD graphene functionalized with molecule **8**$^{3+}$ in positive (left side) and negative ion mode (right side) ion mode. Both, the mass of the cation (m/z = 513.28, left side) and the mass for the counterion (m/z = 148.95) were found on the whole tested sample surface, supporting the overall homogeneous functionalization of CVD graphene. Surface scan area: 500 µm x 500 µm.

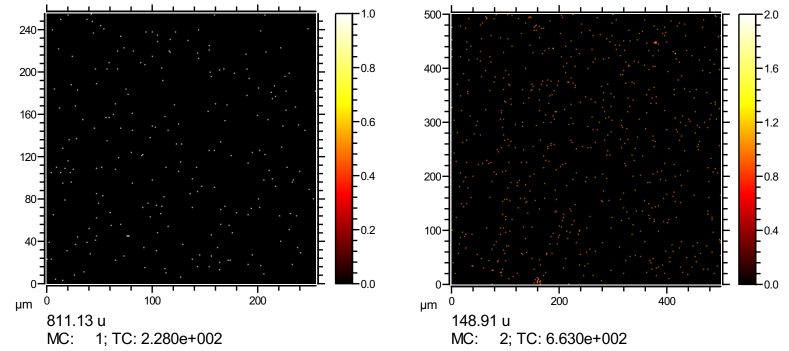

Figure S38. ToF-SIMS images of a silicon wafer, which was incubated in a methanolic solution of trication molecule **8**$^{3+}$ in positive (left side) and negative ion mode (right side) ion mode, showing neither the mass of the cation (m/z = 811.20, left side) nor the mass for the counterion (m/z = 148.95) on the tested sample surface. This indicates that trication 8 is not interacting with the silicon surface. Surface scan area: 500 µm x 500 µm.

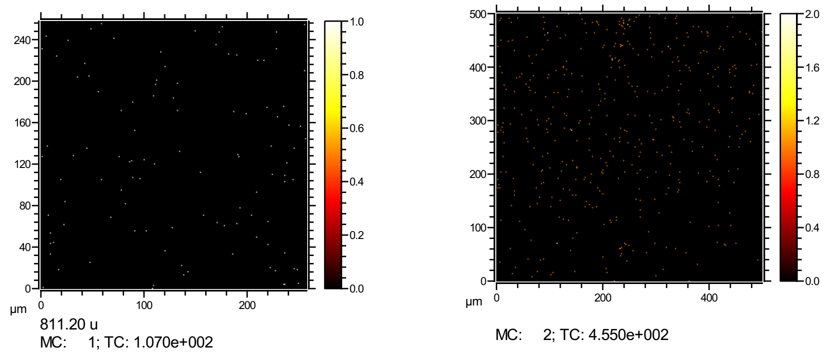

Figure S39. ToF-SIMS images of a silicon wafer, which was incubated in methanol in positive (left side) and negative ion mode (right side) ion mode. Neither the mass of the cation (m/z = 811.20, left side) nor the mass for the counterion (m/z = 148.95) was found on the tested sample surface. Surface scan area: 250 µm x 250 µm.

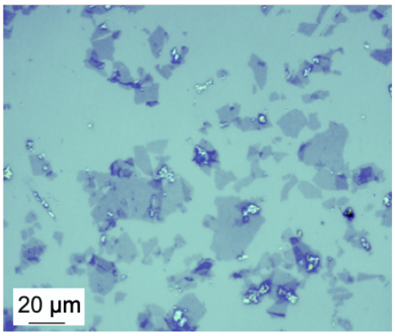

Figure S40. Optical image of r-oxo-G flakes functionalized with molecule **8**$^{3+}$. The flake size varies between 5 and 20 µm.

Atomic Force Microscopy

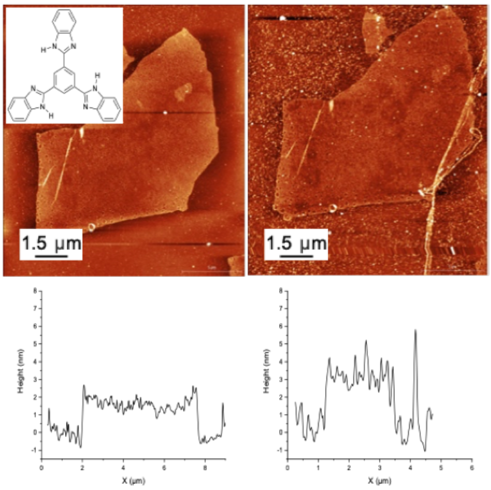

Figure S41. AFM image of reduced-oxo-G (left side, top) and AFM image of r-oxo-G functionalized with molecule **4** (right side, top). High profile of reduced-oxo-G (left side, bottom) and high profile of r-oxo-G functionalized with molecule **4** (right side, bottom).

Discrete Fourier Transform Calculations

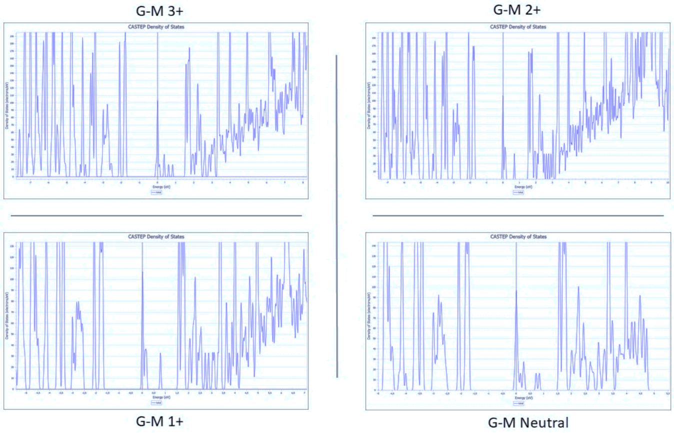

Figure S42. a) Details of the densities of States for G-M complexes with different charges. Notice the similar structures around the Fermi level.

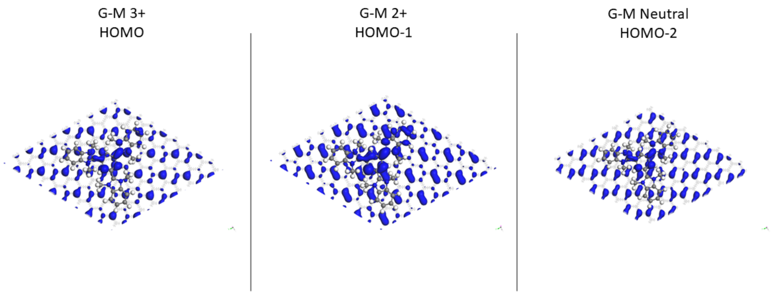

Figure S43. a) Relevant orbitals for the charged G-M complexes.

In as much as DFT predicts electron transfer from graphene to tricationic molecule **6**$^{6+}$, further signatures for such reduction of ionic charge, from **8**$^{3+}$ to **8**$^{2+}$, is provided here. Thus, Raman spectra for **8**$^{3+}$, **8**$^{2+}$, and **8**$^{1+}$ in the gas phase have been computed. Indeed, it is the assumed non-covalent nature of the bonding between cation and graphene that implies changes in the Raman spectra of the molecular ions in the gas phase to be relevant. The qualitative differences in computed spectra allow the further validation of the non-covalent bonding. Hence, support for single-electron transfer is arrived at by matching the experimental Raman spectra of tricationic molecule **8**$^{3+}$ (**Figure 4A**) in solution to the computed Raman spectrum of the **8**$^{3+}$ charged moiety **6**$^{3+}$ in the gas phase (**Figure S41**), and tricationic molecule **8**$^{3+}$ on CVD graphene **8**$^{3+}$ (**Figure 4B**) to the computed Raman spectrum of **8**$^{2+}$, while excluding the **8**$^{1+}$.

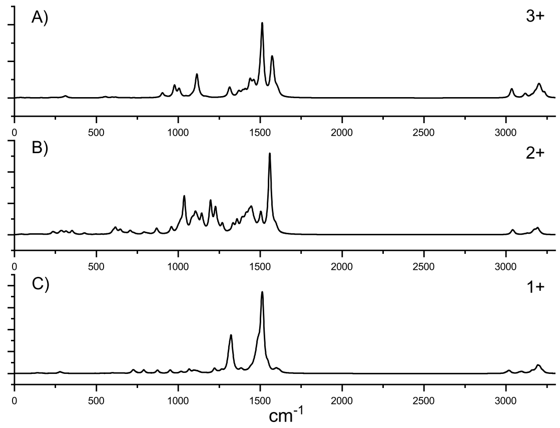

Figure S44. Calculated Raman spectra of A) trication molecule **8**$^{3+}$, b) the reduced species **8**$^{2+}$ and C) the twice reduced species **8**$^{1+}$ on graphene.

Cyclic Voltammetry characterization of 9^{3+}:

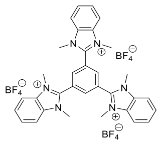

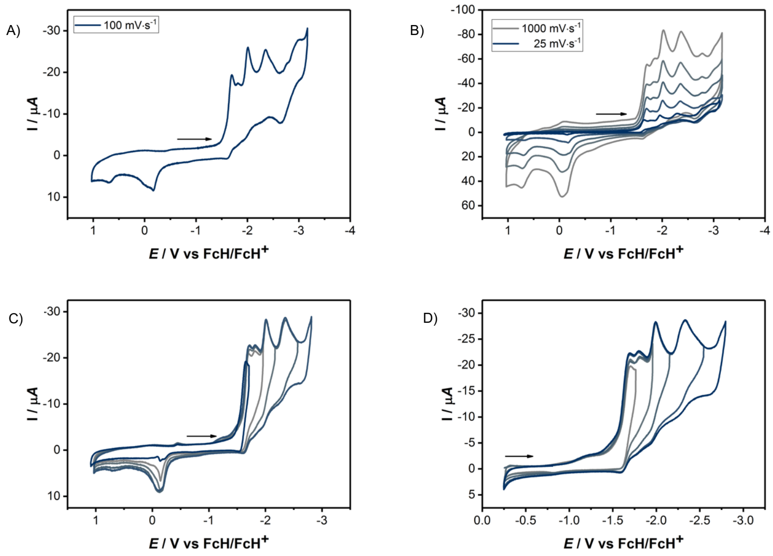

Figure S45. Cyclic voltammetry of $\mathbf{9^{3+}}$ in ACN (+0.1 M Bu_4NPF_6) at different scan rates. A) Overview scan of $\mathbf{9^{3+}}$ at 100 mVs^{-1} and B) overview scans of $\mathbf{9^{3+}}$ at different scan rates between 25 - 1000 mVs^{-1}. C) Overview scans of $\mathbf{9^{3+}}$ at 100 mVs^{-1} showing the dependency of the oxidation peak from each reduction event. D) Overview scans of $\mathbf{9^{3+}}$ at 100 mVs^{-1} showing the reduction events without the oxidation event.

Cyclic Voltammetry characterization of 12^{3+}:

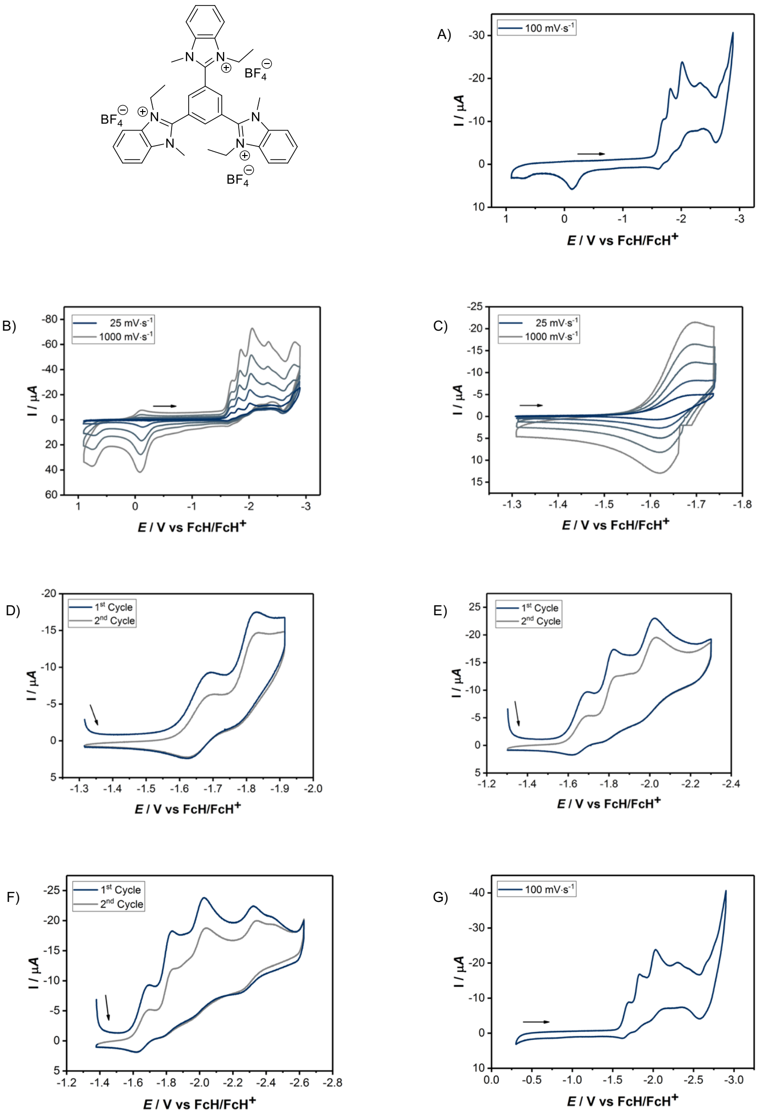

Figure S46. Cyclic voltammetry of **12^{3+}** in ACN (+0.1 M Bu_4NPF_6) at different scan rates. A) Overview scan of **12^{3+}** at 100 mVs^{-1} and B) overview scans of **12^{3+}** at different scan rates between 25 - 1000 mVs^{-1}. C) Cyclic voltammogram of first reduction peak from **12^{3+}** at $E_{red,1}$ = -1.65 V at different scan rates between 25 - 1000 mVs^{-1}. D) Cyclic voltammogram of first and second reduction events from **12^{3+}** at 100 mVs^{-1}. The second cycle (grey line) is showing adsorption on the electrode surface. E) Cyclic voltammogram of first, second and third reduction events from **12^{3+}** at 100 mVs^{-1}. The second cycle (grey line) is showing adsorption on the electrode surface. F) Cyclic voltammogram of first, second, third and fourth reduction events from **12^{3+}** at 100 mVs^{-1}. The second cycle (grey line) is showing adsorption on the electrode surface. G) Overview scan of **12^{3+}** at 100 mVs^{-1} showing the reduction events without the oxidation event.

UV-Vis-NIR spectroelectrochemistry of 12^{3+}:

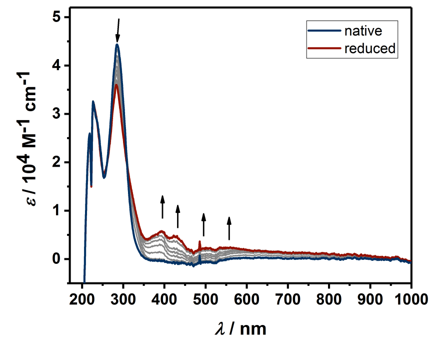

Figure S47. Changes in the UV-vis-NIR spectrum of **12**$^{3+}$, during first reduction.

Table S1. In-situ spectroscopy for the native species (**12**$^{3+}$)

Wavelength [nm]	Extinction coefficient [$M^{-1}\cdot cm^{-1}$]
218	25710
227	32550
285	44370

Table S1. In-situ spectroscopy for the reduced species (**12**$^{3+}$)

Wavelength [nm]	Extinction coefficient [$M^{-1}\cdot cm^{-1}$]
218	25710
227	32550
285	35880
393	2785
423	4885
506	2270
553	2360

Transport measurements:

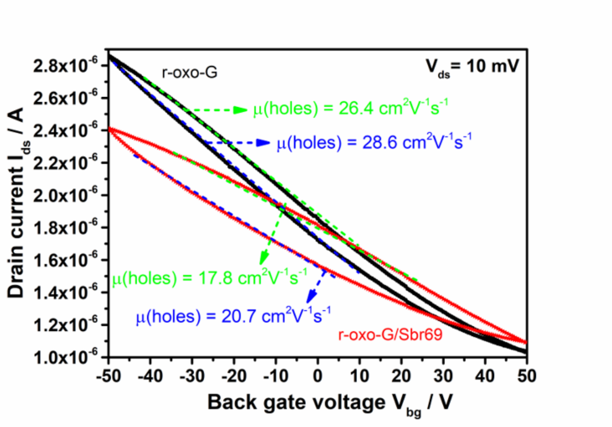

Figure S48. Comparison [illegible] nes) for r-oxo-G (black) and r-oxo-G/**8**$^{3+}$ (red, r-oxoG/SBr69).

We calculated each two carrier mobilities for r-oxo-G and r-oxo-G/**8**$^{3+}$ (**Figure S48**). The carrier mobilities were based on the minimum (blue dash lines) and the maximum slopes (green dash lines) of both curves. For the r-oxo-G, the fluctuation of its carrier mobilities is between 26.4 and 28.6 $cm^2V^{-1}s^{-1}$. After functionalization with tricationic molecule **8**$^{3+}$, the carrier mobilities of r-oxo-G/**8**$^{3+}$ are between 17.8 and 20.7 $cm^2V^{-1}s^{-1}$. Obviously, the effect of hysteresis on carrier mobilities is less than that of r-oxo-G/**8**$^{3+}$. Based on this point, we can also confirm that the effect of air on the sample can be negligible.

References

[1] N. Chandrashekhar, B. Thomas, V. Gayathri, K. V. Ramanathan, N. M. Gowda, *Magn. Reson. Chem.* 2008, 46, 769-774.

[2] W. K. Fife, P. Ranganathan, M. Zeldin, *J. Org. Chem.* 1990, 55, 5610-5613.

[3] C. K. Chua, M. Pumera, *Chem.: Eur. J.* 2013, *19*, 2005-2011.

[4] S. Eigler, M. Enzelberger-Heim, S. Grimm, P. Hofmann, W. Kroener, A. Geworski, C. Dotzer, M. Rockert, J. Xiao, C. Papp, O. Lytken, H. P. Steinrück, P Müller, A. Hirsch, *Adv. Mater.* **2013**, *25*, 3583-3587; b) S. Eigler, A. Hirsch, *Angew. Chem. Int. Ed.* **2014**, *53*, 7720-7738.

[5] S. Grimme, J. Comput. Chem. 2004, 25(12), 1463-1473.

[6] M. Krejčik, M. Daněk, F. Hartl, *J. Electroanal. Chem. Interfacial Electrochem.* 1991, *317*, 179-187.
